# Of mice and men: translating mouse knockout models of human male infertility

**DOI:** 10.1530/REP-25-0260

**Published:** 2025-09-08

**Authors:** Seleen Barada, Marwa Alameer, Reem H Aldhaheri, Hamdan Hamdan, Junaid Kashir

**Affiliations:** ^1^ Department of Biological Sciences, College of Medicine and Health Sciences, Khalifa University, Abu Dhabi, United Arab Emirates; ^2^ Food Security and Technology Center, Khalifa University, Abu Dhabi, United Arab Emirates

**Keywords:** male infertility, genetics, spermatogenesis, mouse models, assisted reproductive technology (ART), reproduction

## Abstract

Infertility is a global health issue affecting a significant portion of couples, estimated at ∼10–15% of reproductive-aged couples worldwide, with the World Health Organisation (WHO) suggesting roughly one in six (∼17.5%) of the adult population experiences infertility. Male causes of infertility are attributable as a sole or leading cause in 40–50% of cases. Furthermore, sperm/semen counts have plummeted by ∼60% over the past 50–60 years in males attending fertility clinics. There is thus an urgent need to understand the causes behind these numbers to address such worrying trends. However, human male infertility is a heterogeneous and often idiopathic condition, with genetic factors increasingly recognised as major contributors. In this review, we examine known and emerging genetic causes of male infertility, highlighting how knockout mouse models have been leveraged to understand not only male reproductive biology and sperm physiological function, but also to illustrate how specific genetic disruptions correspond to particular reproductive failures, discussing how such mouse models are illuminating the causes of human idiopathic male infertility and guiding the discovery of novel infertility genes. We compare the similarities and differences between human and mouse infertility, not only identifying areas of further investigation that require urgent attention, but also potential novel avenues of therapeutic treatment.

## Introduction

Infertility is a global health issue affecting a significant portion of couples, estimated to affect ∼10–15% of reproductive-aged couples worldwide, with the World Health Organization (WHO) suggesting approximately one in six (∼17.5%) of the adult population experiences infertility (Cox *et al.* 2022, WHO 2023). Such figures seem constant throughout regions and income levels, with infertility rates numbering ∼17.8% in high-income vs ∼16.5% in low- and middle-income countries, indicating that infertility is a growing universal rather than socio-cultural challenge (WHO 2023). Clinically defined as the inability to achieve a pregnancy after 12 months or more of regular unprotected intercourse, the burden of infertility is considerable, underlying profound emotional distress and social stigma. Infertility also affects couples financially, increasingly representing a significant financial strain on couples seeking fertility treatment (Njagi *et al.* 2023).

Epidemiologically, ∼20–35% of infertility cases are attributable to female factors alone, ∼20–30% to male factors alone, and ∼25–40% to both partners (combined factors). A large portion of the remaining 10–20% of cases is unexplained (idiopathic) (Agarwal *et al.* 2015). Male infertility is attributable as a sole or leading cause in 40–50% of cases (Schlegel *et al.* 2021). However, this varies regionally, with male infertility accounting for 20–70% of cases depending on the region observed, with the highest rates of male factor infertility observed in African and Eastern European countries (Agarwal *et al.* 2015). Indeed, ∼7% of all men globally are estimated to be infertile (Kasman *et al.* 2020). Specifically, male fertility seems to be undergoing a rapid global decline, with sperm/semen parameters declining by ∼60% over the past 50–60 years in males attending fertility clinics (Levine *et al.* 2017, Sciorio *et al.* 2024). There is thus an urgent need to understand the causes behind such numbers to perhaps reverse, but more importantly treat, such worrying trends.

Mouse genetic knockout (KO) models have long served as a cornerstone for elucidating gene function in reproduction, offering a powerful framework for interrogating the molecular basis of spermatogenesis, sperm function, fertilisation, and fertility. In this review, we build upon many highly detailed reviews that discuss a range of genetic mutations linked to human infertility, and also integrate recent high-throughput findings with established genetic data from mouse models to classify infertility phenotypes and explore their translational relevance. We examine the physiological processes involved in key aspects of spermatogenesis, spermiogenesis, and fertilisation, and identify recent novel mutations that contribute to our understanding of these processes. Expanding upon this, we also highlight critical gaps, particularly where species-specific differences limit clinical extrapolation, and propose strategic directions for refining the use of genetic models in uncovering human infertility causes.

## Causes of male infertility

**Figure 1:**
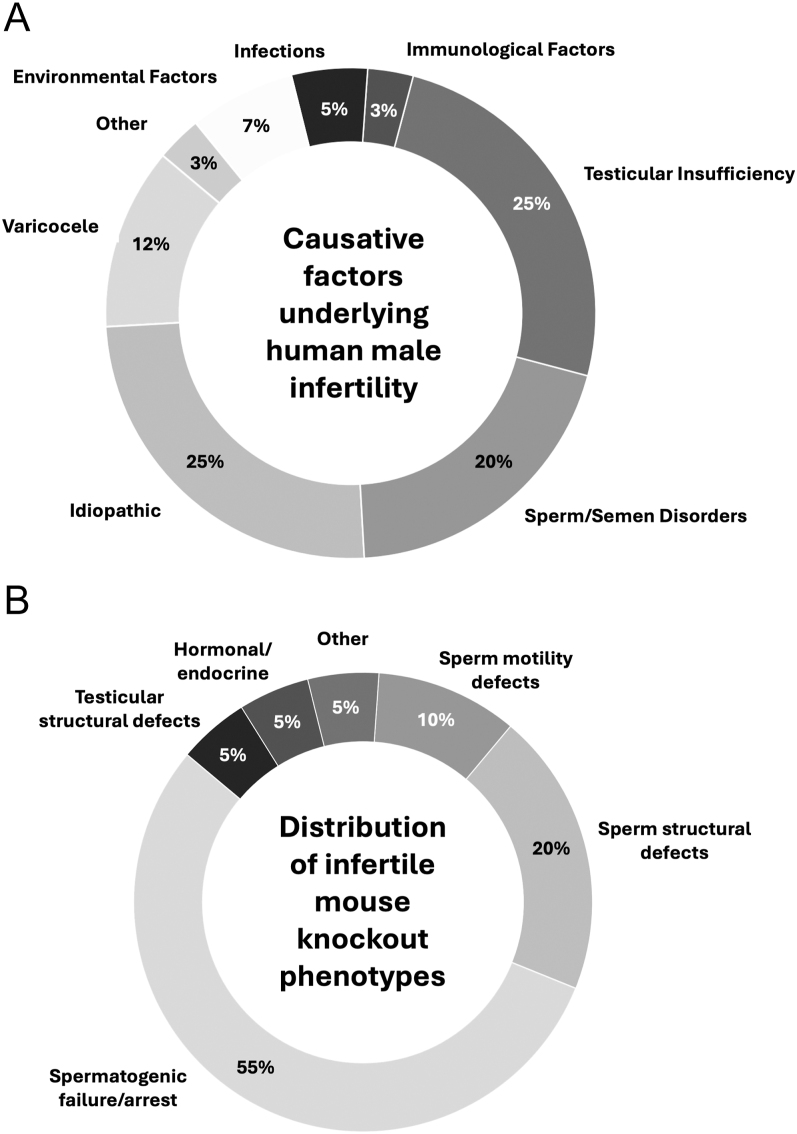
Comparative overview of aetiological contributors to human infertility and relative phenotypes of mouse infertility knockout models. (A) Pie chart illustrating the distribution of causative factors underlying male infertility in humans. Data are expressed as estimated percentages derived from clinical and epidemiological studies, highlighting the predominance of idiopathic cases and varicocele, alongside genetic, endocrine, immunological, and environmental contributions. Distributions compiled from Alhathal *et al.* (2020), Kashir *et al.* (2010). (B) Proportional distribution of phenotypic classifications among infertile knockout mouse models, based on the current literature. Most mouse models exhibit spermatogenic failure or arrest (55%), followed by defects in sperm structure (20%) and motility (10%), with additional contributions from testicular, hormonal, and miscellaneous abnormalities. Data were compiled by performing a search for strains on the Mouse Genomics Informatics (MGI) database (https://www.informatics.jax.org/; last accessed 3rd July 2025) using ‘male’ and ‘infertility’ as keywords. Mouse information data were then collated into the various groups based upon keyword characterisation (testicular structural defects: ‘testicular’, ‘cryptorchid’, ‘microrchid’, ‘gonadal dysgenesis’, and ‘ectopic testis’; hormonal/endocrine: ‘hypogonadism’, ‘hormone’, ‘FSH’, ‘LH’, ‘androgen’, ‘pituitary’, ‘GnRH’, and ‘endocrine’; sperm motility defects: ‘motility’, ‘astheno-‘, ‘dyskinesia’, ‘flagella’, ‘immotile’, and ‘multiple morphological abnormalities of the sperm flagella’; sperm structural defects: ‘acrosome’, ‘head’, ‘tail’, ‘terato-‘, ‘morphological abnormality’, ‘centriole’, and ‘globozoospermia’; spermatogenic failure/arrest: ‘spermatogenic failure’, ‘azoospermia’, ‘oligozoospermia/oligospermia’, and ‘meiotic arrest’; other: entries that did not match any of the above heuristics; Nagirnaja *et al.* 2018). Further data were also compiled from Ogorevc *et al.* (2011) using the same characterisations and keywords. Data compiled from (Kashir *et al.* 2010, Ogorevc *et al.* 2011, Nagirnaja *et al.* 2018, Alhathal *et al.* 2020).

Male infertility is a multifactorial condition influenced by a range of underlying causes (Fig. 1A), including anatomical defects such as varicocele, vesicular damage from torsion, blockages in the testicular sperm passage, and ejaculatory dysfunction. Other factors include genital tract infections, gametogenesis issues, genetic disorders, hormonal imbalances, and immune-related problems (Matzuk & Lamb 2008, Varghese *et al.* 2008, Rivero *et al.* 2023). For clarity, considering that most of these causes can be attributable to an underlying genetic cause (or a combinatorial effect of multiple genetic factors), the current review classifies infertility conditions based on the phenotypic outcomes, and where appropriate discusses further the associated genes involved. In addition, a man’s fertility is significantly influenced by lifestyle and environmental factors. For example, weight gain and smoking have been associated with problems in gamete and embryo development (Bocu *et al.* 2024, Rotimi & Singh 2024).

A significant portion of male infertility remains idiopathic, meaning that no definitive cause can be attributed, and it is typically diagnosed after excluding all known factors. Such men exhibit abnormalities in sperm/semen parameters, or exhibit infertility with an unexplained aetiology, representing ∼25–30% of male infertility cases, especially when including men with mild to moderate abnormalities with no clear origin (García-Baquero *et al.* 2020, Boeri *et al.* 2024). Without specific attributable causes, managing such cases is challenging. Such figures underscore the need for continued research to uncover subtle or complex factors that may explain such cases. A large role in this has been played by the establishment of genetic KO mouse models, which have illuminated several panels of genes that seem essential for male fertility (significantly more than in females) (Singh & Schimenti 2024).

The Mouse Genome Informatics (MGI) database lists 286 genes that produce reproductive system phenotypes in mice, 147 of which are also present in both humans and mice (Ogorevc *et al.* 2011, Nagirnaja *et al.* 2018). Large-scale phenotyping efforts and targeted CRISPR screens have also systematically tested fertility in knockout lines, confirming that >400 genes yield male infertility (Alhathal *et al.* 2020) (Fig. 1B), of which only a few have been investigated in detail (Supplementary Table 1 (see section on Supplementary materials given at the end of the article)).

## Spermatogenic arrest/failure and testicular abnormalities

It is clear that most male-sterile mouse KO models exhibit primary defects in sperm production, specifically failures in spermatogenesis, with smaller subsets exhibiting isolated sperm functional defects or endocrine abnormalities (Nagirnaja *et al.* 2018, Alhathal *et al.* 2020). Phenotypes include loss of germ cells (Sertoli cell-only seminiferous tubules), failure of spermatogonia differentiation, or meiotic arrest (blockage during meiosis I or II), resulting in azoospermia (no sperm) or severe oligozoospermia. Numerous gene KO models disrupted meiosis, causing apoptotic loss of spermatocytes. Some cause early germ cell loss, such as a Sertoli-cell-only syndrome (complete absence of germ cells), which accounts for ∼15% of azoospermic cases in humans. Overall, spermatogenic failure (arrest at a pre-sperm stage) is the predominant outcome, accounting for roughly 50–60% of infertile KO phenotypes.

**Figure 2:**
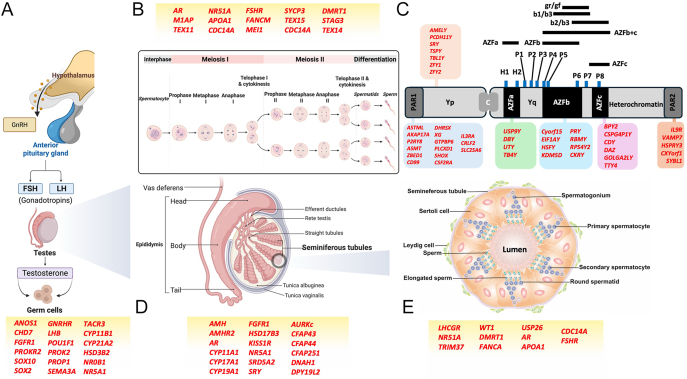
Genetic regulation of male reproductive function across the hypothalamic–pituitary–gonadal (HPG) axis, meiotic cell divisions, Y chromosome regions, testicular and seminiferous tubule architecture. (A) Overview of the hypothalamic–pituitary–gonadal (HPG) axis showing hormonal control of testicular function. Gonadotropin-releasing hormone (GnRH) from the hypothalamus stimulates the anterior pituitary to release luteinising hormone (LH) and follicle-stimulating hormone (FSH), which act on the testes to regulate testosterone production and spermatogenesis. Key genes (red text) involved in germ cell regulation and testicular development include key transcription factors and hormonal regulators. (B) Schematic indicating meiotic progression during spermatogenesis from spermatocyte, through meiosis I and II, to haploid round spermatids, followed by differentiation into sperm. Genes associated with meiotic arrest and differentiation failure are shown in red. (C) Schematic representation of the Y chromosome, focussing on regions (AZFa, AZFb, and AZFc) implicated in male infertility due to microdeletions. Genes located within and flanking the azoospermia factor (AZF) regions are displayed in relation to known Y-linked spermatogenic failure phenotypes (red text). (D) Schematic representation of mammalian testicular and epididymal organisation in relation to genes (red text), which when disrupted have been linked to abnormalities in structure. (E) Organisation of seminiferous tubules, each of which is composed of a basal lamina embedded within interstitial tissue that contains Leydig cells, blood vessels, and immune cells. Within the seminiferous epithelium, germ cells develop in a stratified manner, progressing from undifferentiated spermatogonia at the basal compartment towards mature elongated spermatids at the luminal edge. Sertoli cells span the full thickness of the epithelium, providing physical and regulatory support to developing germ cells. The two compartments are separated by the blood–testis barrier (BTB), a tight junction complex formed between adjacent Sertoli cells. The organisation of seminiferous tubules is critical for the temporal and spatial regulation of spermatogenesis, and disruption of this architecture is a hallmark of many forms of male infertility, particularly in relation to the genes identified in red text. (A, B, and D) Created in BioRender. Kashir, J (2025) https://BioRender.com/4lze2s6.

Included in this category are testicular abnormalities caused by a somatic or structural defect in the testis architecture rather than within germ cells, and thus are classed separately from spermatogenic arrest/failure, as such conditions would result from defects in testicular architecture rather than spermatogenic cells. ∼5% of male-infertility models were primarily due to such structural defects, with phenotypes including testicular malformation (dysgenesis) and Sertoli-cell-only syndrome, where the testis tubules lack germ cells entirely. A smaller subset of KO models resulted from endocrine or hormonal disruptions, which impaired fertility. These include genes in the hypothalamic–pituitary–gonadal axis or hormone receptors required in the testis (Fig. 2A). Classic examples are KO of gonadotropins or their receptors, where KO males were unable to produce testosterone. Overall, although hormone pathway knockouts represent only ∼3–5% of male-infertility models, they underscore the necessity of hormonal cues for fertility (Sengupta *et al.* 2021).

Spermatogonial stem cells (SSCs) initially go through mitotic divisions to increase the stem-cell pool and produce progenitor cells that mark the transition towards differentiation. Sertoli cell-derived growth factors, particularly glial cell line-derived neurotrophic factor (GDNF) induced by follicle-stimulating hormone (FSH), further regulate differentiation of type A spermatogonia to type B spermatogonia, which will enter meiosis as primary spermatocytes (Cannarella *et al.* 2020). Primary spermatocytes then cross the blood-testis barrier and enter the adluminal compartment of the seminiferous tubules where they undergo meiosis I stages. Multiple molecular factors are expressed throughout this process to ensure proper chromosome segregation, double-stranded break (DSB) repair, and crossing over (Cannarella *et al.* 2020). Meiosis II then follows, producing haploid round spermatids by splitting sister chromatids.

Successful meiosis completion is a key driver for the production of mature, fertile sperm, resulting in the production of haploid gametes (oocytes or sperm) (Feng *et al.* 2014). In spermatogenesis, chiasmata formation is critical to ensure proper homologous chromosome segregation during the first meiotic division. This homologous interaction during pairing is facilitated by a telomere-led rapid prophase chromosome movement, and initiates with the formation of programmed DNA DSBs that are continuously repaired by homologous recombination to complete the meiotic prophase (Kauppi *et al.* 2013).

Meiosis itself is further composed of two stages, I and II. The first stage is the reduction phase, where chromosome number is halved by a choreographed alignment and accurate segregation of homologous chromosomes. However, meiosis II resembles mitosis due to the absence of further chromosome number reduction, and focuses on separating sister chromatids (Handel & Schimenti 2010). The first stage (prophase I) ensures high-fidelity segregation through at least one crossover between each pair of homologous chromosomes (Baudat & de Massy 2007). This crossover is facilitated by programmed meiotic DSBs, predominantly between the leptotene and zygotene stages (de Massy 2013). Approximately 200–300 DSBs are created at specific locations in the genome known as recombination hotspots (Baudat *et al.* 2013).

The primary regulator of the formation and location of these hotspots in mammals is PR domain-containing protein 9 (*PRDM9*), a meiosis-specific histone H3 methyltransferase produced between the preleptotene to pachytene stages. *PRDM9* is involved in protein interactions and transcriptional repression, the trimethylation of histone H3 at lysines 4 and 36 (H3K4me3 and H3K36me3), and sequence-specific DNA binding (Grey *et al.* 2018), recognising specific DNA motifs and guiding the placement of epigenetic marks to remodel chromatin. While the zinc finger domain of *PRDM9* alters rapidly across species and individuals, leading to variation in the positioning of recombination hotspots, the same variability can also cause reproductive problems. *PRDM9* is currently the only known gene in vertebrates that directly causes hybrid sterility, confirmed by studies using mice bred from wild subspecies (Gregorova *et al.* 2018). Deletion of *Prdm9* resulted in meiotic arrest, leading to both male and female infertility (Hayashi *et al.* 2005), largely thought to be due to abnormal DSB formation and impaired chromosome pairing (synapsis). In *Prdm9*-KO mice, although nearly 99% of the usual DSB hotspots were altered, ∼94% of new DSBs still occurred at sites marked by H3K4me3, a histone modification associated with active gene regions (Brick *et al.* 2012).

Spermiogenesis follows the completion of meiosis, where spermatids mature into spermatozoa. No cellular division occurs, but critical changes include acrosome formation, nuclear elongation and condensation, flagellum formation, and removal of excess cytoplasm (Tanaka & Baba 2005). DNA is packaged with protamines that replace histones to compact the DNA, essential to silence sperm transcriptionally. After these changes, spermatozoa are produced that then leave the testes (Cannarella *et al.* 2020) (Fig. 2B). Following spermiogenesis completion in the testes, spermatozoa lack motility and fertilising capability. To acquire these features, sperm undergo further post-testicular maturation, beginning with epididymal maturation in the male, followed by capacitation in the female reproductive tract (Gervasi & Visconti 2017). Epididymal maturation requires the spermatozoa to transit in the highly segmental epididymis, composed of the initial segment (proximal to the testis), caput, corpus, and cauda (which connects to the vas deferens) (Sullivan *et al.* 2019). These distinct epididymal segments impart changes that encompass the incorporation of new molecules secreted by the epididymal epithelium and post-translational modifications of proteins synthesised earlier during spermiogenesis (Gervasi & Visconti 2017).

Due to the high level of idiopathic male infertility cases, it is essential to understand how spermatogenesis and downstream processes could be affected, causing human infertility. Spermatogenic failure is an aspect of this, which can be caused by disruptions in the sperm genome and other factors such as epigenetic disruptions, obesity, diabetes, environmental factors, and varicocele (enlargement of spermatic veins). Klinefelter syndrome (KS) is the most common genetic cause of male infertility and is characterised by an extra X chromosome in males. This is speculated to mainly be caused by nondisjunction during meiosis I, where the homologues fail to separate properly. KS impacts spermatogenesis by progressively degenerating germ cells and Sertoli cells. Y-chromosome microdeletions within the azoospermic factor (AZF) region, which holds genes essential for spermatogenesis, could impair this process. MicroRNAs are a class of small RNA molecules that have been shown to affect protein expression by inhibition, which could affect the presence of necessary spermatogenesis players. In addition, epigenetic factors such as DNA methylation could alter gene expression without changing the DNA sequence, thus affecting the transcription of spermatogenesis protein-coding genes (Neto *et al.* 2016, Cannarella *et al.* 2020) (Fig. 2C).

Several KO models have allowed the identification of master regulatory factors that control broad aspects of spermatogenesis. *DAZL* is an excellent example, which functions as a master translational regulator essential for multiple stages of sperm development throughout vertebrate germ cells, and is essential for embryonic germ cell development and differentiation. Conditional KO of *Dazl* in mouse germ cells caused complete male sterility, with a gradual loss of spermatogonial stem cells, meiotic arrest, and spermatid arrest (Li *et al.* 2019). *DAZL* seems to control spermatogenesis via direct translational regulation rather than transcriptional control, as when *DAZL* is absent, polysome-associated target transcripts decreased significantly while total transcript levels remained unchanged, associated with drastic reductions in spermatogenic proteins and subsequent developmental arrest (Fig. 2D and C).

This further suggests that RNA processing is also a vital component of spermatogenesis. *CWF19L2* represents a good example, given its status as a fundamental component of the spliceosome, regulating alternative splicing. *Cwf19l2*-KO in germ cells caused spermatogenesis failure and complete male infertility (Wang *et al.* 2024*a*). *Cwf19l2* also interacted with several spliceosome proteins, including *Pabpc1*, *Hnrnpm*, *Ddx5*, *Dhx9*, *Prpf8*, and *Prpf43*, participating in spliceosome assembly and stability. Furthermore, *Cwf19l2* directly bound and regulated splicing of genes related to spermatogenesis (*Znhit1*, *Btrc*, and *Fbxw7*) and RNA splicing (*Rbfox1*, *Celf1*, and *Rbm10*) (Wang *et al.* 2024*a*), demonstrating how single factors control complex regulatory networks.

### Azoospermia

∼15% of infertile men are diagnosed with an absence of spermatozoa in the ejaculate, also known as azoospermia. Clinically, this can be further differentiated into two classes based on aetiology: obstructive azoospermia (OA) and non-obstructive azoospermia (NOA) (Tekayev & Vuruskan 2021). OA is characterised by physical blockage in any region of the male reproductive ductal system between the rete testis and the ejaculatory ducts (Jow *et al.* 1993). Conversely, NOA is attributed to primary testicular failure (elevated LH, FSH, small testes affecting up to 10% of men presenting with infertility), secondary testicular failure (congenital hypogonadotropic hypogonadism with decreased LH and FSH, small testes), or incomplete or ambiguous testicular failure (either increased FSH and normal volume testes, normal FSH and small testes, or normal FSH and normal testis volume) (Wosnitzer *et al.* 2014).

NOA testicular biopsies typically exhibit substantial histological variation, typically classified into three groups: Sertoli cell-only (SCO), complete maturation arrest (MA), and mixed atrophy. The difference in the histopathology of the testis might be the consequence of the complexity of the spermatogenesis process and its variable genetic and epigenetic regulation (Gershoni *et al.* 2017). Any errors in the meiotic processes throughout spermatogenesis could result in defective spermatogenesis and NOA. Specifically, some defects within the meiotic recombination processes result in meiotic arrest, which causes sterility (Handel & Schimenti 2010). Single-gene defects have largely been associated with key genes and NOA. A major example includes X-linked *TEX11*, disruptions in which have been associated in men with complete meiotic arrest and NOA. Strikingly, *Tex11*-KO mice exhibit almost identical phenotypes of meiotic failure and infertility (Yatsenko *et al.* 2015), with similar observations for other meiosis-critical genes such as *Stag3*, *Msh5*, and *M1ap* (Jiao *et al.* 2021).

NOA can also result from hormonal imbalances that disrupt testicular function, particularly within the hypothalamic–pituitary–gonadal (HPG) axis. Gonadotropin-releasing hormone (GnRH), secreted by the hypothalamus, stimulates the anterior pituitary to release luteinising hormone (LH) and follicle-stimulating hormone (FSH) (Kaprara & Huhtaniemi 2018). LH acts on Leydig cells to promote testosterone production, while FSH targets Sertoli cells, stimulating the production of inhibin B and androgen-binding globulin (ABG) and supporting the development of germ cells (Mäkelä *et al.* 2019). Together, these gonadotropins initiate and maintain spermatogenesis and regulate testicular hormone production. Early reports had suggested that men with mutations in the FSH receptor (*FSHR*) could still be fertile (Tapanainen *et al.* 1997). However, more recent research indicates that when FSH activity is completely absent, it leads to azoospermia (Zheng *et al.* 2017). This apparent contradiction was explained by the discovery that some mutated FSHRs retain minimal residual function (Rannikko *et al.* 2002). In contrast, mutations in the FSHβ subunit gene (*FSHB*) result in complete FSH deficiency due to disrupted hormone–receptor interactions (Zheng *et al.* 2017). Human cases of isolated *FSHB* mutations are rare and typically present with azoospermia despite normal testosterone levels. *Fshb*-KO mice exhibited a less severe phenotype. *Fshr*-KO mice displayed impaired Sertoli cell function and testicular development, yet remained fertile under laboratory conditions, suggesting species-specific differences or compensatory mechanisms not present in humans (Sairam & Krishnamurthy 2001).

Luteinising hormone (LH) plays a central role in regulating male reproductive function by stimulating Leydig cells to produce testosterone, which in turn acts on Sertoli cells to support spermatogenesis (Kaprara & Huhtaniemi 2018). Mutations affecting LH signalling lead to isolated LH deficiency and male infertility. In humans, loss-of-function mutations in the LH β-subunit gene (*LHB*) resulted in low testosterone, undermasculinisation, cryptorchidism, micropenis, and azoospermia, even when FSH levels remain normal. These individuals typically require exogenous LH or hCG therapy to induce puberty and initiate spermatogenesis. *Lhb*-KO mice exhibited undetectable testosterone, cryptorchidism, and disrupted spermatogenesis, closely mimicking the human phenotype. Likewise, LH receptor (*Lhcgr*)-KO mice exhibited Leydig cell aplasia, low testosterone, and infertility (Kaprara & Huhtaniemi 2018).

## Sperm structural/morphological defects

The head compartment of sperm contains the nucleus and the acrosome needed to penetrate the oocyte’s zona pellucida (ZP). The acrosome is a cap-like structure that houses digestive enzymes required to allow for oocyte penetration and is formed from remnants of the Golgi apparatus during spermiogenesis. The sperm flagellum is a specialised motile cilium with an axoneme primarily featuring a microtubule 9 + 2 pattern. The sperm annulus is an important ring-like structure that separates the midpiece (MP) of the flagellum, just below the head, from the principal piece (PP), which is composed of various proteins, including Septin polymers (SEPT 2, 4, 6, 7, and 12) (Al-Ali *et al.* 2024) and the Testis Anion Transporter 1 (*SLC26A8*) (Whitfield 2024).

The flagellum is made up of multiple components that regulate sperm movement, mainly through two dynein arms (inner dynein arm (IDA) and outer dynein arm (ODA)), which function as ATP-driven motors attached to the microtubules. A nexin-dynein regulatory complex (N-DRC) coordinates the movements of the dynein arms and links the microtubules together (Azhar *et al.* 2021). A radial spoke (RS) mechanical structure also connects the outer microtubule doublets to the central pair to regulate movement through the dynein motors. A calmodulin- and spoke-associated complex (CSC) is another regulatory complex of the dynein motors that bridges the RS, the N-DRC, and the IDA and uses calmodulin as a calcium (Ca^2+^) regulatory sensor. Other structures of the axoneme, namely the peri-axoneme, include the outer dense fibres (ODFs), the fibrous sheath (FS), and the mitochondrial sheath (MS), with the fibrous sheath housing glycolytic enzymes and signalling molecules (Zhou *et al.* 2024*b*).

Following spermatogenesis and spermiogenesis, sperm are transported to the epididymis, where they undergo further changes to acquire primary motility via activation of signalling and metabolic pathways. Multiple protein phosphorylation cascades have been associated with this process, such as the WNT, GSK3 kinase, and PPP1 and PPP2 phosphatases. Low bicarbonate (HCO3^−^) levels in the environment of the epididymis are needed to prevent premature capacitation and allow the sperm to be stored in a quiescent state within the caudal region of the epididymis before ejaculation. Once ejaculated, sperm complete their final maturation step through biochemical changes known as capacitation within the female reproductive tract. This process focuses on hyperactivation of the sperm that enables them to navigate through the oviduct, increased membrane polarisation and fluidity, cytoplasmic alkalinisation, and the activation of PKA through Ca^2+^ and HCO3 levels and calmodulin-dependent kinase signalling pathways that phosphorylate proteins within the flagellum. These processes ultimately enable the sperm to achieve maximum motility through increases in amplitude and velocity of flagellar beating and allow the sperm to penetrate the ZP of the oocyte and achieve fertilisation (Puga Molina *et al.* 2018, Cavarocchi *et al.* 2022).

Such KO models exhibited sperm production but with abnormal morphology (head or flagellum defects) that impeded function. KO models primarily affecting sperm structure and morphology exhibit phenotypes including teratozoospermia (abnormal sperm shape) such as globozoospermia (round-headed sperm lacking acrosomes) or flagellar malformations. These structural deficits often lead to non-viable or immotile sperm despite completion of spermatogenesis. Globally, ∼20% of known male-sterile lines can be attributed to primary sperm morphological abnormalities. A number of KO models exhibited deficiencies in sperm tail structural proteins (axonemal dyneins and fibrous sheath components) resulting in malformed or short flagella and male sterility.

### Teratozoospermia

Teratozoospermia is a condition characterised by morphologically abnormal sperm, with two major subtypes: acephalic spermatozoa syndrome (ASS) and globozoospermia (a specific subtype of teratozoospermia characterised by a round head and lack of an acrosome). ASS is a rare but severe form exhibiting headless sperm or defects in the formation of the head-tail coupling apparatus (HTCA) during spermiogenesis (Jiao *et al.* 2021). Key proteins involved are *SAD1* and UNC48 domain containing 5 (*SUN5*/*SPAG4L*), which localise at the sperm head-tail junction. *SUN5* mutations affect nearly half of all ASS cases in humans, with *Sun5*-KO mice exhibiting a complete acephalic sperm phenotype (Shang *et al.* 2017). Similarly, polyamine modulated factor 1 binding protein 1 (*PMFBP1*) mutations have been identified in humans, and *Pmfbp1*-KO mice are infertile with acephalic sperm due to disrupted coupling between the sperm head and tail (Zhu *et al.* 2018). Both proteins are well linked to ASS; the list expands to other proteins that have been mentioned in association due to their effects on the HTCA.

*Ccdc42*-KO resulted in HTCA malformation alongside multiple morphological anomalies of the flagella (MMAF) phenotypes. *Ccdc188*-KO mice were also infertile with ASS (Qiu *et al.* 2025). Mutations in CFAP52 were found in ASS patients, and *Cfap52*-KO mice exhibited a mix of MMAF and ASS phenotypes (Jin *et al.* 2023). *CFAP52* also interacts with spermatogenesis-associated protein, *SPATA6*, a structural protein within the HTCA, which has been associated with ASS through loss-of-function studies but has yet to be identified in ASS patients (Yuan *et al.* 2015). Hook microtubule tethering protein 1 (*HOOK1*) is essential for the assembly of the manchette, an array of microtubules, and its attachment to the nucleus. Mouse *Hook1*-KO mice, commonly referred to as *azh*/*azh* mice, exhibit an abnormally formed manchette and misshaped sperm head (Mendoza-Lujambio *et al.* 2002). However, a corresponding mutation in humans has yet to be identified. Centrosomal proteins (CEP), such as *CEP112*, were mutated in some ASS patients, although their mechanism is not understood, and KO mice studies have not been performed (Zhang *et al.* 2024).

Globozoospermia is characterised by abnormalities or absence of the acrosome in the sperm head, originating during spermiogenesis. It is characterised by round-headed sperm and has several key genetic causes (Azhar *et al.* 2021). Human genes that have been largely associated with globozoospermia include proteins interacting with C kinase 1 (*PICK1*), *SPATA16*, zona pellucida binding protein 1 (*ZPBP1*), and Dpy-19-like-2 (*DPY19L2*). *Pick1*-KO mice exhibited disrupted acrosome formation and globozoospermia phenotypes due to failed vesicle trafficking between the Golgi apparatus and the acrosome, leading to a lack of acrosome synthesis (Xiao *et al.* 2009). *Zpbp1*-KO showed its importance in acrosome formation, as resulting mice were infertile with globozoospermia (Lin *et al.* 2007). Similarly, *DPY19L2* anchors the acrosome to the nucleus of the sperm head, with *Dpy19l2*-KO resulting in globozoospermic mice with absent acrosome anchoring during biogenesis and, eventually, its absence (Pierre *et al.* 2012). *SPATA16* is noteworthy, as a mutation was found in three human patients with globozoospermia. However, 29 other patients with the same condition did not exhibit this mutation. *SPATA16* is highly expressed in the testes and is understood to be required for the fusion of the Golgi apparatus with the vesicles involved in acrosome formation. However, *Spata16*-KO demonstrated a lack of spermiogenesis instead of globozoospermia (Fujihara *et al.* 2017). Sperm Acrosome Associated 1 (*SPACA1*) is a membrane protein localised to the acrosomal section and is thought to be involved in acrosome formation and sperm/oocyte fusion, as anti-SPACA1 antibody could inhibit human sperm fusion with zona-free hamster oocytes (Hao *et al.* 2002). *Spaca1*-KO mice displayed phenotypes associated with globozoospermia, specifically a lack of an acrosome. Interestingly, *Zpbp1*-KO mice also lacked SPACA1 protein (Fujihara *et al.* 2012).

### Multiple morphological anomalies of the flagella (MMAF)

Multiple morphological anomalies of the flagella (MMAF) is a type of severe astheno-teratozoospermia that affects the morphology of the flagella and, consequently, the motility of the sperm. Although this term was only proposed in 2014, the phenotypes in male sperm were thought to be associated with another condition called primary ciliary dysplasia (PCD) due to the similar defects observed in sperm tails. However, MMAF has gained independence as a distinct condition for male infertility without other clinical symptoms. Diagnosis typically requires that >5% of sperm show at least four distinct tail abnormalities, including short, coiled, absent, or irregularly shaped flagella (WHO 2021), resulting in immotile and structurally impaired sperm. At the structural level, MMAF results in dysfunction within the axoneme and peri-axonemal (surrounding structures supporting the axoneme) structures. In place of the typical flagella structure, MMAF can cause disorganised microtubule components, frequently missing microtubule doublets and/or central pairs, and potentially even lacking dynein arms, including inner dynein arms (IDA) and outer dynein arms (ODA). Peri-axonemal disorganisation can include malformed/missing outer dense fibres, disorganised fibrous sheaths, and impaired mitochondrial sheath. Zhou *et al.* (2024*b*) have extensively reviewed the genetic factors relating to MMAF mutations and their corresponding mouse models.

ODAs and IDAs are essential to move flagellar oscillations to propel the sperm forward properly. Cilia- and flagella-associated proteins (CFAP) play diverse roles in flagellar structure, involving the ODAs, the IDAs, and the radial spoke (RS). The dynein heavy chain (DNAH) proteins play a crucial role in the flagellar structure of sperm, particularly within the inner and outer dynein arms. *Dnah*-KO can disrupt the regulation of dynein arm movements, leading to classical MMAF phenotypes (Liu *et al.* 2020, Zhang *et al.* 2021*a*). DNAH and associated factors also play critical roles in maintaining the IDAs, with KO of these genes also showing MMAF phenotypes. *DNAH1* was classified as the first gene associated with MMAF abnormalities, contributing to about 24.6% of MMAF cases (Wang *et al.* 2020*a*). The list of IDA-implicated genes has since expanded to include more DNAH proteins such as DNAH2, DNAH6, DNAH7, and DNAH10, and other CFAP proteins such as CFAP43 and CFAP44; dynein heavy-chain domain 1 (DNHD1), also known as coiled-coil domain-containing 35 (CCDC35), and WD repeat-domain 63 (WDR63). DNAH1 serves as an anchor for the RS and was interestingly found to interact with dynein axonemal light chain 1 (DNAL1) in an IDA subcomplex (Ben Khelifa *et al.* 2014, Wu *et al.* 2023). Similarly, *Dnal1*-KO mice displayed complete infertility while maintaining normal morphology (Hwang *et al.* 2021, Tu *et al.* 2021, Wu *et al.* 2023, Wang *et al.* 2024*b*).

Another important group of proteins within IDA structures are cilia- and flagella-associated proteins (CFAPs), such as CFAP43 and CFAP44, which localise adjacent to IDAs and are implicated in approximately 30.8% of MMAF patients. *Cfap44*-KO male mice were infertile and exhibited severe sperm motility and morphological impairment. *Cfap44*-KO mice displayed similar phenotypes, with a slight discrepancy compared to human patients with these variants, where mice exhibited somewhat fewer flagellar defects (Tang *et al.* 2017). A *Wdr63*-KO model of the WDR63 protein that functions as part of the IDA intermediate chain exhibited both the MMAF phenotype and oligozoospermia (low sperm count) in mice (Lu *et al.* 2021). Other models, such as *Dnhd1*-KO male mice, showed typical MMAF phenotypes. DNHD1 is part of the CCDC protein family, with several members significantly implicated in MMAF, and has emerged as a critical regulator of the sperm flagellum (Tan *et al.* 2022). Dynein regulatory complex subunit 1 protein (DRC1) has also been found to cause MMAF phenotypes, and *Drc1*-KO mice showed that DRC1 is essential for the assembly of N-DRC and resulted in impaired sperm motility (Zhang *et al.* 2021*b*).

Other proteins implicated in MMAF are linked to the RS, which consists of RS1 (adjacent to the IDA), RS2 (connected to the N-DRC), and RS3. The complex formed between the RS and the CSC is crucial for the overall assembly and stability of sperm flagella, with any changes leading to structural abnormalities. Some proteins that can influence this interaction include CFAP61, CFAP206, CFAP65, and CCDC146. Additional proteins associated with MMAF via the RS include Adenylate kinase 7 (AK7), CFAP91, CFAP251, and CCDC189, although they have not yet been characterised in human and mouse subjects (Zhou *et al.* 2024*b*). Removal of CSC proteins impacts the integrity of this complex and subsequently affects morphology and motility due to disorganisation of the microtubule structure, a disrupted flagellum, disrupted manchette organisation, and disrupted interaction with other proteins (Li *et al.* 2020, Muroňová *et al.* 2024).

The Sperm Flagellar 2 gene, *SPEF2*, was one of the few proteins found to be associated with the central microtubules within the sperm flagella. Its variance in humans and *Spef2*-KO mice models showed MMAF phenotypes such as MS, ODFs, and FS scattering (Tu *et al.* 2020). In addition, fibrous sheath-interacting protein 2 (FSIP2) is a key component of the fibrous sheath, along with A-Kinase Anchoring Protein 4 (AKAP4), also part of the fibrous sheath, and Glutamine-rich protein 2 (QRICH2), which serves as a glutamine sensor for regulation. Knock-in/KO mouse models for these proteins exhibited MMAF phenotypes, characterised by abnormal flagellar structures, impaired motility, and infertility (Fang *et al.* 2019, Fang *et al.* 2021). TBC1D21, a protein that plays a role in mitochondrial organisation and axonemal assembly, KO models of which resulted in infertile male mice due to impaired sperm flagella structure and mitochondrial defects, with diminished sperm motility. *TBC1D21* defects have been implicated in patients with human teratozoospermia (morphological defects only). However, KO mice also identified diminished sperm motility, so the classification of this protein cannot yet be entirely attributed to MMAF phenotypes in humans (Wang *et al.* 2020*b*).

## Sperm motility defects

A further subset of KO models produced sperm that were morphologically intact but functionally immotile or poorly motile (asthenozoospermia-AZS). ∼10% of male-infertility models fall in this group as a primary phenotype (although there is significant overlap with the group exhibiting structural defects). These cases often involve genes required for flagellar beating, sperm axoneme function, or sperm metabolism. KO models of several ciliary/flagellar proteins (e.g. DNAH family dynein heavy chains, the cation channel sperm-associated, CatSper, calcium channel subunits) lead to immotile sperm with normal morphology (in the case of *CatSper*-KO this is specifically loss of hyperactivated motility). Furthermore, many axoneme and motility gene KO models also resulted in male sterility due to the same reasons. We note that some models show combined mild morphology and motility defects (often grouped as oligo-astheno-teratozoospermia), but here we count those primarily affecting motility. Sperm acquire motility throughout spermiogenesis, alongside concurrent morphological changes within haploid, round-shaped spermatids as they develop into mature, elongated spermatozoa. This process involves nuclear condensation and the rounding of the sperm head, as well as flagellar assembly, which is an integral component necessary for sperm motility.

### Asthenozoospermia (AZS)

Proteins underlying sperm structure contribute significantly towards dysfunction in sperm motility, leading to AZS. Functional AZS arises from abnormalities in sperm without significant morphological defects and involves gene defects associated with signalling pathways that affect primary motility and capacitation. This can include ion transporters and channels, energy metabolism processes, and some cytoskeletal effects. Cavarocchi *et al.* (2022) have extensively reviewed animal models of sperm ion transporters and channels, which highlight potentially key players such as the CatSper family, SLC26 transporters, voltage-dependent anion channels (VDAC), and SLO3 potassium channels, all of which are integral to the success of capacitation. Defects in these channels and transporters affect Ca^2+^ signalling, pH modulation, and the hyperpolarisation of the sperm membrane, which lead to reduced sperm motility and, in some cases, morphological defects (Cavarocchi *et al.* 2022). Of significant note are recent findings that the CatSper channel in mice functions as a temperature-gated channel, with a thermal threshold of 33.5°C. Furthermore, this temperature gating is reversibly inhibited by spermine (a seminal plasma protein), ensuring prevention of premature channel activation (Swain *et al.* 2025). Although similar observations remain to be made in humans, this does provide intriguing directions towards the potential role of other factors that indirectly affect major ion channels involved in sperm motility.

Additional gene mutations affecting sperm energy metabolism also result in impaired motility, and thus, asthenozoospermia. Energy metabolism within sperm is a crucial process that generates sufficient ATP to enable their motility throughout the female reproductive tract. Factors such as adenylate kinase 9 (AK9), which catalyses the conversion of ADP to ATP and AMP, impact sperm motility when knocked out in mouse models (Sha *et al.* 2023*a*). Glycolysis and mitochondrial oxidative phosphorylation (OXPHOS) are the primary pathways that produce ATP for sperm motility. Interestingly, mitochondrial DNA (mtDNA) encodes some of the subunits involved in OXPHOS. Therefore, specific gene deletions in mtDNA that disrupt essential genes have been identified as risk factors for AZS in patients; however, mouse models are unavailable for suggested genes, most encoding electron transport chain protein complexes (Cavarocchi *et al.* 2025).

In terms of cytoskeletal effects, the variants of these genes reduce sperm motility. However, depending on the extent of morphological defects, some begin to overlap with AZS. Septins, for example, are a family of cytoskeletal proteins most attributed to their role in cell division. However, they are also associated with spermatogenesis. *Sept4*-KO mice exhibited bending of the flagella at the MP/PP junction and an absent annulus (Kissel *et al.* 2005). Other factors, including TEKT3, IQUB, KIF9, and DNAL1, influence cytoskeletal dynamics and have been studied through KO models that exhibited reduced sperm motility (Roy *et al.* 2009, Miyata *et al.* 2020, Wu *et al.* 2023, Zhang *et al.* 2023).

### Oligoasthenoteratozoospermia (OAT)

OAT is a complex condition characterised by a combination of multiple factors. Patients with OAT exhibit low sperm counts, reduced motility, and abnormal morphology. An acrosomal membrane protein, MFSD6L, interacts with SPACA1, with mutations identified in OAT patients. *Mfsd6l*-KO resulted in subfertile mice with OAT and caused the loss of proteins crucial for fertilisation and oocyte activation (Zhou *et al.* 2024*a*). Another Ccdc protein, CCDC157, is associated with OAT, KO of which led to male infertility in mice by disrupting the function of the Golgi apparatus and acrosome biogenesis, resulting in OAT phenotypes. The loss of CCDC157 also caused downregulation of vesicle formation and trafficking genes (such as *Pick1*) and acrosomal matrix genes (such as *Spaca1*) (Zheng *et al.* 2024). KO of terminal nucleotidyltransferase 5D (*Tent5d*), a protein essential for maintaining mRNA stability and translation during spermiogenesis, impaired RNA processing and stability, leading to irregular regulation of spermiogenesis, resulting in infertile mice displaying OAT (Sha *et al.* 2023*b*).

Tudor domain-containing proteins (TDRD) are also crucial for the piRNA pathway, which silences RNA transposable elements, ensuring genomic stability and proper sperm development. *Tdrd*-KO mice also exhibited OAT phenotypes (Bi *et al.* 2024). Another CFAP protein, CFAP61, has previously been associated with MMAF, but seems also to have further connections to OAT (Hu *et al.* 2023). A *Cep135* conditional KO mouse exhibited OAT phenotypes in infertile males; however, human patients with mutations in *CEP135* were linked to the MMAF phenotype (Liu *et al.* 2025*a*). Conversely, CEP78 defects demonstrated OAT in humans and mice, and the *Cep78*-KO model exhibited extremely low sperm count, abnormal morphology, and nearly no sperm motility, hypothesised to result from differences between mutations and a complete KO (Liu *et al.* 2025*b*).

## Other/miscellaneous causes

**Figure 3:**
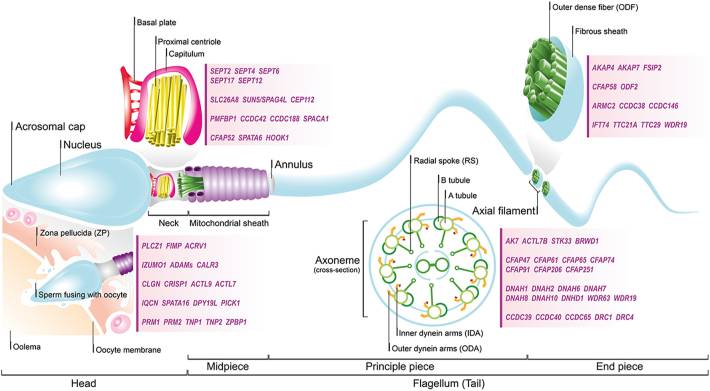
Schematic illustration of the molecular architecture of mammalian sperm, highlighting genes implicated in structural and functional infertility. The major structural regions of sperm are represented (head, midpiece, flagellum (tail)), with key genes annotated at their respective subcellular locations based on their known functions and associations with male infertility. The head contains genes critical for oocyte interaction, fertilisation, and acrosome/nuclear integrity. The midpiece, responsible for energy production and structural connectivity, features genes associated with annulus formation and mitochondrial sheath organisation. The mitochondrial sheath is subdivided into the axoneme, further consisting of radial spokes (RS), and outer/inner dynein arms (ODA/IDA). Genes involved in flagellar assembly, motility, and morphology, many of which are linked to multiple morphological abnormalities of the sperm flagella (MMAF), are localised to components of the sperm flagellum, and include genes that regulate microtubular integrity, dynein motor activity, and flagellar stabilisation. Gene names are positioned relative to their functional compartments in the schematic within shaded boxes.

This set of KO models (∼5–10%) includes genes whose loss caused infertility through less typical or systemic pathways, with many involved in genome integrity, chromatin dynamics, or cellular metabolism, with their KO models often manifesting as infertility alongside pleiotropic effects. For example, DNA damage response gene KO models often caused male infertility due to high germ cell apoptosis rates from unrepaired DNA breaks (Gunes *et al.* 2015). Similarly, chromatin regulator abnormalities led to failed fertilisation or early embryonic arrest despite normal sperm counts (Spanò *et al.* 2000, Sadeghi *et al.* 2009, Tesarik & Mendoza Tesarik 2025). Other miscellaneous factors include autoimmunity phenotypes, post-testicular defects (e.g. epididymal blockage), or metabolic deficiencies affecting sperm function/motility (Fig. 3).

### DNA fragmentation

A factor which has gained prominence with regards to human infertility is a phenomenon termed sperm DNA fragmentation (SDF), referring to the presence of single- or double-stranded breaks in the DNA of sperm cells (Agarwal *et al.* 2022). Unlike traditional semen parameters – sperm concentration, motility, and morphology – SDF relates to the genomic integrity of sperm, which plays a pivotal role in fertilisation, embryo development, implantation, and pregnancy success (Avendaño *et al.* 2010, Speyer *et al.* 2010). Notably, approximately 40% of infertile men present with normal semen parameters according to WHO criteria, yet may still exhibit high levels of DNA damage that compromise fertility (Agarwal *et al.* 2020). The causes of SDF are multifactorial, including abortive apoptosis, defective protamination during chromatin condensation, and oxidative stress due to excess reactive oxygen species (ROS), with additional contributing factors including varicocele, advanced paternal age, smoking, genital infections, chemotherapy, and poor lifestyle habits (Esteves *et al.* 2021, Andrabi *et al.* 2024). Given such wide-reaching overlap between other infertility syndromes and DNA fragmentation, it is perhaps understandable that there is a paucity of KO mouse models directly examining DNA fragmentation in mice. However, there are key studies which identify potential models that could enhance our understanding.

Protamination is a unique chromatin remodelling process in which histones are first replaced by transition nuclear proteins (TNPs) and subsequently by protamines (PRMs) during the final stages of spermiogenesis, a stepwise transition that is highly conserved across mammals including mice and humans (De Vries *et al.* 2012). KO mouse models of the two PRMs (*Prm1* and *Prm2*) although capable of producing sperm, failed to sire offspring. Even a reduction in the amount of either PRM resulted in defective nuclear formation and impaired sperm function (Cho *et al.* 2001). This corresponds to findings where *Prm1* gene variants correlated to male infertility, where patients exhibited high levels of SDF (Aydos *et al.* 2018), with a similar role suggested for *Prm2* variants (Yang *et al.* 2016). Similarly, the functional relevance of TNPs has been validated, where although individual KO models of *Tnp1* and *Tnp2* were subfertile, double KO models were completely infertile (Yu *et al.* 2000, Zhao *et al.* 2001, Zhao *et al.* 2004).

Finally, given the role played by ROS in eliciting SDF, enzymes that play key roles in regulating such a phenomenon have also received scant attention. For example, glucose-6-phosphate dehydrogenase (G6PD) is a key enzyme in the hexose monophosphate shunt responsible for producing NADPH, which maintains cellular antioxidant defences that protect sperm from ROS (Stanton 2012, García-Domínguez *et al.* 2022), and thus against SDF. *G6pd*-KO male mice exhibited increased oxidative damage and SDF, poor fertilisation outcomes, and elevated embryonic loss (García-Domínguez *et al.* 2022), with similar observations for corresponding mutations in humans (Wu *et al.* 2014).

### Total fertilisation failure (TFF) and oocyte activation deficiency (OAD)

Until now, we have discussed aspects of male infertility that result in sperm structural defects, resulting in either absent sperm or severely impaired structure that underlies male infertility. However, in the modern era of assisted reproduction, most cases can be treated with the advent of intracytoplasmic sperm injection (ICSI; whereby a single cell is injected directly into the oocyte). Indeed, ICSI can be used to successfully treat NOA following testicular sperm retrieval via methodologies such as microsurgical testicular sperm extraction (TESE). TESE-ICSI, although associated with lower fertilisation, clinical pregnancy, and live birth rates compared to OA and non-azoospermic patients, can result in successful pregnancies and live births, offering some hope to a large portion of NOA men (Majzoub *et al.* 2024), even if such outcomes are controversial. Similar views are available for morphologically abnormal sperm, such as globozoospermic sperm, whereby ICSI (with some further intervention) can be used to successfully achieve pregnancy and live birth (Crafa *et al.* 2023), with a similar outlook for asthenozoospermia (Chen *et al.* 2022), teratozoospermia (Hotaling *et al.* 2011), oligozoospermia (Esteves 2022), and OAT (Jiang *et al.* 2024). However, there are populations of men whose sperm, despite exhibiting relatively normal morphology, are unable to result in successful fertilisation, even following ICSI. Total fertilisation failure (TFF) is defined as the inability of sperm to fertilise mature metaphase II (MII) oocytes, even after ICSI. Oocyte activation deficiency (OAD) is a significant cause of TFF, characterised as the oocyte’s inability to undergo oocyte activation. TFF affects around 1–3% of ICSI cycles, and can occur despite normal sperm morphology, motility, and concentration (Campos *et al.* 2023).

Oocyte activation (OA) in mammals is initiated by repetitive transients in intracellular levels of calcium (Ca^2+^ oscillations), mediated by delivery of a soluble sperm factor, largely accepted to be phospholipase C zeta (PLCZ1) via the inositol 1,4,5-trisphosphate (IP3) pathway (Kashir *et al.* 2012*b*). These oscillations are specific and unique to each species, and further initiate a series of molecular events, including meiosis II resumption, second polar body extrusion, cortical granule exocytosis, and pronuclear formation, making PLCZ1 integral to fertilisation and embryogenic success (Swann 2025). Abnormalities in PLCZ1 structure, expression, and localisation underlie cases where oocyte activation is deficient (OAD) (Nomikos *et al.* 2017). Indeed, numerous mutations in *PLCZ1* have been associated with human OAD, with several corresponding mouse KO models assessed. Sperm from *Plcz1*-KO mice were unable to elicit Ca^2+^ release following injection into oocytes, while exhibiting severely high polyspermy, reduced patterns of Ca^2+^ release, and significantly reduced litters only following *in vitro* fertilisation (IVF) (Hachem *et al.* 2017, Nozawa *et al.* 2018). Furthermore, Kashir *et al.* (2024) also demonstrated that although *Plcz1*-KO sperm could result in fertilisation following IVF, the resultant embryogenic profile was abnormal and resulted in severely impaired rates of embryogenesis.

A few other factors could also contribute to TFF or OAD. One such factor is sperm head decondensation failure, whereby the male pronucleus fails to fuse with the female pronucleus, attributable to poor sperm chromatin packaging or DNA damage (Kashir *et al.* 2018). Although classically attributed to oocyte-related factors (Campos *et al.* 2023), defects in chromatin packaging via protamines, sperm DNA fragmentation, or improper sperm head condensation have been linked as integral to this process. However, specific genes or groups of proteins have yet to be identified in the context of humans.

A key component of TFF is sperm/oocyte fusion. Some key proteins, including IZUMO1, the ADAM family of proteins, CALR3, CLGN, and CRISP1, are involved in the initial recognition and binding of sperm to the zona pellucida (ZP) and then the oolemma. ADAM proteins facilitate sperm migration through the oviduct and binding to the oocyte ZP. Mice lacking ADAM2 and ADAM3 via KO models were infertile (Han *et al.* 2010). The calreticulin protein (CALR3) is a chaperone crucial for the maturation of ADAM3. CALR3 absence results in sperm which, despite appearing normal with standard motility, are ineffective in migrating through the oviduct and ZP binding, leading to TFF (Ikawa *et al.* 2011). IZUMO1 is a sperm transmembrane protein integral to the binding and fusion of sperm and oocyte through its corresponding oocyte receptor protein, JUNO. *Izumo1*-KO mice were completely infertile, and were unable to fuse with the oolemma despite exhibiting ZP penetration (Inoue *et al.* 2005). However, human mutations associated with *IZUMO1* have not yet been found. Another sperm protein, Fertilisation Influencing Membrane Protein (FIMP) – a testis-specific transmembrane protein – is critical for sperm–oocyte fusion independently of IZUMO. However, similar to IZUMO, such factors have not yet been associated with specific mutations in humans.

While not directly related to OAD, other factors are involved in maintaining proper sperm head structure and function, as well as the localisation and expression of sperm factors involved in mediating TFF. Actin-like 9 (ACTL9) and Actin-like 7 (ACTL7) are located in the perinuclear theca (PT) beneath the acrosome, and are crucial for oocyte activation, perinuclear theca structure, and acrosome anchoring. *Actl9*-KO and *Actl7a*-KO mouse models exhibited structural acrosomal defects, alongside abnormal PLCZ1 levels and localisation (Dai *et al.* 2021, Ferrer *et al.* 2023). Furthermore, structurally related proteins such as SPATA16, DPY19L, and PICK1, although associated with globozoospermia, might also play a role in TFF. IQCN is a protein localised to the acrosome that functions as a scaffold, and infertile patients exhibiting *IQCN* biallelic mutations experience fertilisation failure and sperm head abnormalities even after ICSI. *Iqcn*-KO mice exhibited acrosomal defects including PLCZ1 mislocalisation, and an acrosomal matrix protein (ACRV1) being disrupted. These effects led to fertilisation failure in mice through IVF and ICSI due to the inability of the acrosome reaction to occur (Sha *et al.* 2023*b*).

## Prospects for clinical treatment

Knowledge gained from associations between human male infertility and mouse KO models are increasingly allowing for translation to diagnostics and patient management (Jiao *et al.* 2021). Indeed, several fertility clinics now offer gene panel testing or whole exome sequencing for men exhibiting idiopathic infertility, aiming to identify hidden monogenic causes. Although the current diagnostic prospect of such testing remains limited to within animal models, even a slight incremental gain could yield a considerable increase in fertility treatment success. For example, use of a curated gene panel in genetic testing identified causal mutations in ∼2% of men with unexplained severe oligo/azoospermia, improving the ∼10–15% yield of traditional tests such as karyotyping and Y-chromosome microdeletion analysis (Podgrajsek *et al.* 2025).

Such advances can further enhance patient care, enabling improved genetic counselling and potentially sparing patients from invasive procedures required for further diagnosis. For example, if a patient exhibits a deleterious mutation linked to meiotic arrest also validated in KO mouse models, clinicians can avoid interventions such as testicular sperm extraction (TESE) surgeries, with the couple instead being counselled on alternative options, such as the use of donor sperm or adoption (Podgrajsek *et al.* 2025). Such advances also stand to be further enhanced given recent advances in gene editing technologies. Methods such as CRISPR/Cas-mediated systems or conditional KO models have greatly enhanced the speed of model creation and study of gene function at specific developmental stages or cell types. Such precision highlights how some genes have multiple functions at various developmental stages, providing insights that would be impossible to obtain from traditional KO approaches alone.

As the catalogue of known infertility genes continues to grow, the scope of such genetic diagnostics will expand, being added to diagnostic panels and thereby gradually increasing the sensitivity of genetic testing. In the short term, this would mean an increased chance of obtaining a definitive molecular diagnosis for previously unexplained infertility, presenting significant psychological and clinical benefits. In some cases, understanding underlying genetic defects could also lead to tailored treatment approaches. A clear example of this is globozoospermia, where standard fertility treatment often fails as the sperm is unable to penetrate or activate the oocyte. Several genes are now associated with this condition, as discussed, as well as a key association with abnormal PLCZ1 in such sperm (Kashir *et al.* 2016, Kashir *et al.* 2020). With this knowledge, ICSI combined with assisted oocyte activation (through use of Ca^2+^ ionophores such as A23187) has successfully resulted in fertilisation, pregnancy, and delivery to term (Kashir *et al.* 2022, Abdulsamad *et al.* 2023).

Beyond such immediate applications, growing insights from mouse KO models also lay the groundwork for potential future fertility treatments, whereby targeted interventions can be developed to restore or compensate for certain defects. Indeed, if a patient’s infertility is linked to a missing protein or hormonal signal, supplementation of that factor or stimulating the appropriate pathway pharmacologically could be an option to consider. In cases where spermatogenic failure is absolute, emerging technologies offer additional hope. One such avenue is *in vitro* gametogenesis, whereby functional gametes are attempted to be generated from patient somatic cells in the laboratory, technology which has been successfully explored in animal models with significant advances in human cells as well (Kashir *et al.* 2012*a*, Murase *et al.* 2024, Singh & Schimenti 2024). Identification of essential genes in these processes will allow build-up of a ‘shopping list’ of essential fertility genes that would be essential to achieving these goals (Singh & Schimenti 2024).

## Differences in mouse and human spermatogenesis

**Figure 4:**
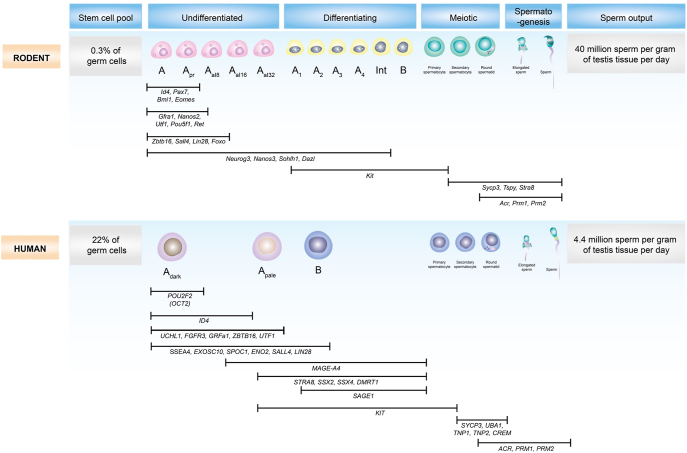
Comparative schematic of spermatogonial differentiation and gene expression dynamics in rodent and human testis, illustrating the stages of spermatogonial development from the stem cell pool to mature spermatozoa in both rodents (top panel) and humans (bottom panel), highlighting key differences in germ cell composition, gene expression, and sperm production efficiency. Rodent spermatogenesis progresses through A_single_, A_paired_, and A_aligned_ undifferentiated spermatogonia, followed by differentiating A_1_–A_4_, intermediate, and type B spermatogonia, leading to meiotic and post-meiotic stages. In contrast, human spermatogenesis involves A_dark_, A_pale_, and type B spermatogonia. Key regulatory genes are indicated beneath each developmental phase, showing species-specific and conserved gene expression patterns across stages. Notable differences include the proportion of germ cells comprising the stem cell pool (0.3% in rodents vs 22% in humans) and the daily sperm output per gram of testicular tissue (∼40 million in rodents vs ∼4.4 million in humans). The timing and duration of expression for genes involved in self-renewal (e.g. *ID4* and *PLZF*), differentiation (e.g. *C-KIT*), and post-meiotic events (e.g. *PRM1* and *PRM2*) are annotated, underscoring molecular divergences and conserved pathways between species. Figure modified from (Bashiri *et al.* 2023).

While significant detail and information have been gained from studying mouse models of infertility, there are several caveats to keep in mind, particularly relating to species-specific differences between mice and humans (Fig. 4). Mouse seminiferous tubules exhibit a highly synchronised wave of spermatogenesis along the seminiferous tubule, with germ cells developing in coordinated cohorts with neighbouring cells often observed at the same developmental stage. However, such patterns are not observed in primates, particularly in humans, whose seminiferous tubules lack this cohesion, whereby at any given location cells at multiple developmental stages will exist, exhibiting a mosaic pattern rather than a single discrete stage (Gartner & Hiatt 2011, de Rooij 2017). Spermatogenesis is longer in humans than in mice, leading to profound differences in proliferation, synchrony of events, and thus overall relative temporal gene expression profiles (de Rooij 2017, Yoshida 2020). One also must consider that the genetic background of most mice is well defined in experiments (usually inbred strains to preserve the genetic architecture), while in humans, the genetic diversity is incomparable.

While expression of essential core genes, such as protamines or meiotic proteins, is conserved, there are notable variances in genes which could be viewed as essential. An example is NGN3, a transcription factor that marks the differentiation of spermatogonia in mice as meiotic commitment occurs. In humans the homologue NEUROG3 is not detected at equivalent stages. Another example is RARG, which is a key regulator of mouse spermatogonia differentiation, differential expression of which determines arrest of meiotic progression. In humans, however, RARG is not differentially expressed in the spermatogonial population (Bush *et al.* 2024). These differences would mean that a knockout of *Ngn3* or *Rarg* in mice would cause clear spermatogenic defects and infertility, while in humans, a similar effect may not be observed. Indeed, not all conserved genes prove essential for fertility. KO of *Tex33*, an evolutionarily conserved gene among vertebrates and initially expressed in the cytoplasm of round spermatids, exhibited normal spermatogenesis, with the first wave of spermiogenesis unaltered with no observable fertility defects (Zhu *et al.* 2020).

Another example is that humans exhibit multiple Y chromosome-linked deleted in azoospermia (*DAZ*) genes that are required for spermatogenesis, deletion/aberration of which results in spermatogenic failure and NOA. However, mice lack Y-linked *Daz* homologues, instead relying on autosomal expression of the *Dazl* gene, deletion of which results in both male and female infertility (Garretson *et al.* 2023, Ou *et al.* 2024). Mice also often exhibit multiple genes or variant isoforms with compensatory effects, while humans exhibit just a single version. For example, mice exhibit two near-identical isoforms of the axonemal dynein heavy chain protein DNAH1, with KO of the major *Dnah1* isoform producing only mild sperm tail abnormalities due to the compensatory effect of the other isoform. Humans exhibit a single DNAH1 isoform, whereby loss of function mutations result in severe MMAF as previously discussed (Khan *et al.* 2021).

Another significant concern is that the functions of factors may vary between the two species. For example, SOX17 is essential for human primordial germ cell formation, while in mice SOX17 is not involved in PGC formation, but rather relies on SOX2, BLIMP1 and PRDM1/4 for this process (Zhang *et al.* 2018, Bush *et al.* 2024). Another example is SOX30 which is a transcription factor required for late mouse spermatogenesis (KO of which results in arrested spermatid development), and although SOX30 is involved in human spermatogenesis, mutations in *SOX30* did not necessarily uniformly result in male infertility due to a uniform mechanism of spermatid developmental arrest, indicating species-specific differences in compensatory mechanisms (Zhang *et al.* 2018, Bush *et al.* 2024). This can also be observed in the case of soluble sperm proteins such as PLCZ1, where loss of activity mutations in humans corresponded to a lack of Ca^2+^ oscillation-inducing ability and male infertility, while *Plcz1*-KO did not result in infertility outright, instead resulting in subfertility with a severely reduced litter size in all cases. However, ICSI of such sperm into oocytes was unable to induce Ca^2+^ release or embryogenesis, indicating perhaps a redundant mechanism specific to mice during sperm/oocyte fusion (Hachem *et al.* 2017, Nozawa *et al.* 2018, Swann 2022, 2025, Kashir *et al.* 2024).

Finally, something to consider is the definition of ‘fertility’ within the literature. In mice, this is measured as the ability to produce a litter, and alterations to the size of this litter. However, considering that the lab conditions and optimised diets mean that mutations that would severely impair sperm quality and litter size would still be able to yield offspring, they would be labelled as ‘fertile’ or ‘sub-fertile’ in the literature, whereas a comparable reduction in humans would result in infertility. To this degree, mouse models may not be able to detect moderate phenotypic effects that would be clinically important to humans. Indeed, mice breed in much larger numbers (average litter sizes of 10–15 pups) compared to the human average of 1–2 at a time. Thus, while a reduction in sperm parameters would result in a reduced litter size of even 2–3 pups (maintaining fertility), a similar reduction in humans would result in complete infertility.

## Conclusion

Mouse knockout models have fundamentally transformed our understanding of male infertility, helping to systematically dissect the normal molecular mechanisms of spermatogenesis. Studies creating and evaluating such KO models have revealed the critical importance of translational regulation, RNA processing, cell survival, and temporal expression/regulation of such pathways in male fertility, establishing a comprehensive framework for understanding reproductive biology. Indeed, the conservation of reproductive processes between mice and humans has enabled direct translation of many findings, leading to improved diagnostic approaches and identification of potential therapeutic targets. However, it remains clear that studies thus far exhibit a heavy bias towards examination of infertility related to spermatogenic failure, representing 55% of KO models examined. Testicular insufficiencies (including spermatogenic failure and testicular abnormalities) account for ∼25% of human male infertility, indicating that examination and understanding of a wider range of processes are essential going forward. Coupled with the fact that numerous distinctions exist between humans and mice, not all findings from such studies may be readily translatable to clinical fidelity.

The expanding catalogue of infertility genes from such models is already paving the way for more comprehensive genetic testing and biomarker discovery, enabling earlier and more precise aetiological classification of infertile patients. Emerging technologies offer promising avenues of pursuit, including gene editing, stem cell-based therapies, and regenerative approaches such as *in vitro* gametogenesis, which hold promise to one day correct or bypass genetic defects. Ultimately, continued interdisciplinary efforts integrating mouse genetics with clinical data and multi-omics will not only expand the compendium of known infertility genes, but also drive the development of targeted interventions, moving us further towards providing effective clinical interventions for the human condition.

## Supplementary materials



## Declaration of interest

The authors declare that there is no conflict of interest that could be perceived as prejudicing the impartiality of the work reported.

## Funding

JK was supported by a faculty start-up grant awarded by Khalifa University (FSU-2023-015).

## Author contribution statement

All authors contributed towards the literature search, quality assessment, data extraction, and interpretation of articles and data, with SB and MA taking the lead with direction from JK. All authors were involved in drafting of the manuscript, and all authors approved the final version of the article.
