# Supplementary material for: Of mice and men: translating mouse knockout models of human male infertility
Source: Reproduction. 2025 Sep 8;170(4):e250260. doi: 10.1530/REP-25-0260 (PMC12558365; doi:10.1530/REP-25-0260)
Supplement: Supplementary file 1 [file supplementary_materials.pdf]

| Gene Name | Gene Full Name | Function | Infertility (I) / Subfertility (S) | Phenotype Condition(s) | Knockout mouse models | Human Mutation |
|---|---|---|---|---|---|---|
| **CATSPER** | Cation Channel Sperm Associated | Hyperactivation | I | Asthenozoospermia | Qi et al.(2007) | Williams et al. (2015) |
| **SLC26A8** | Solute Carrier Family 26 Member 8 | Sperm motility | S | Asthenozoospermia | Touré et al.(2007) | Dirami et al. (2013) |
| **SLC26A3** | Solute Carrier Family 26 Member 3 | sperm maturation and fertilization capacity | S | Asthenozoospermia | El Khouri et al. (2018) | Wedenoja et al. (2017) |
| **SLO3** | Sperm-Specific Potassium Channel | Acrosome formation, mitochondrial sheath assembly, and the function of K+ channels | I | Asthenozoospermia | Santi et al. (2010) | Lv et al. (2022) |
| **VDAC3** | Voltage-Dependent Anion Channel 3 | Sperm motility / Mitochondrial energy dysregulation | I | Asthenozoospermia | Sampson et al. (2001) | Asmarinah et al. (2012) |
| **SLC9C1** | Solute Carrier Family 9 Member C1 | Sperm motility / Capacitation | I | Asthenozoospermia | Wang et al. (2003) | Cavarocchi et al. (2021) |
| **AK9** | Adenylate Kinase 9 | Sperm motility | I | Asthenozoospermia | O'Callaghan et al. (2023) | Sha et al. (2023) |
| **GALTNL5** | Polypeptide N-Acetylgalactosaminyltransferase Like 5 | Sperm motility / acrosome developement | I | Asthenozoospermia | Takasaki et al. (2014) | Hagiuda et al. (2020) |
| **TEKT3** | Tektin 3 | sprm motility / acrosome formation | I | Asthenozoospermia | Roy et al. (2009) | Liu et al. (2023) |
| **IQUB** | IQ Motif and Ubiquitin Domain Containing | sperm motility / radial spoke assembly | I | Asthenozoospermia | Zhang et al. (2022) | Zhang et al. (2023) |
| **KIF9** | Kinesin Family Member 9 | sperm motility / flagellar waveform symmetry | I | Asthenozoospermia | Miyata et al. (2020) | Meng et al. (2023) |
| **ADCY10** | Adenylate cyclase 10 | sperm motility, deficit in cyclic adenosine monophosphate (cAMP) | I | Asthenozoospermia | Esposito et al. (2004) | Akbari et al. (2019) |
| **GAPDHS** | Glyceraldehyde-3-phosphate dehydrogenase, spermatogenic | sperm motility / flagellar structure / glycolytic activity and disrupt ATP production | I | Asthenozoospermia | Miki et al. (2004) | Elkina et al. (2017) |
| **IQCG** | IQ motif containing G | spermiogenesis/ abnormal sperm morphology and immotility | I | Asthenozoospermia | Li et al. (2014) | Harris et al. (2014) |
| **TEKT2** | Tecktin-2 | sperm motility / disruption of axoneme microtubules and mitochondrial abnormalities | I | Asthenozoospermia | Tanaka et al. (2004) | Zuccarello et al. (2008) |
| **Tekt4** | Tecktin-4 | sperm motility | S | Asthenozoospermia | Roy et al.(2007) | Wu et al. (2012) |
| **Spag6** | Sperm associated antigen 6 | sperm motility / abnormal morphology / axonemal structure | I | Asthenozoospermia | Sapiro et al.(2002) | Xu et al. (2022) |
| **PGK2** | Phosphoglycerate Kinase 2 | Sperm motility | I | Asthenozoospermia | Danshina et al. (2010) | Liu et al.(2016) |
| **NSUN7** | Sad1 And UNC84 Domain Containing 4 | Sperm motility | I | Asthenozoospermia | Harris et al. (2007) | Khosronezhad et al. (2015) |
| **PKD** | Polycystic Kidney Disease | Sperm maturation/function | I | Asthenozoospermia (associated with polycystic kidney disease) | Nie et al.(2013) | He et al.(2018) |
| **DNHD1/CCDC35** | Dynein Heavy Chain Domain 1 / CCDC35 (coiled-coil domain-containing protein 35) | Sperm flagella morphological abnormalities | I | Asthenozoospermia (MMAF) | Tan et al. (2022) | Martinez et al. (2023) |
| **WDR63** | WD Repeat Domain 63 | Sperm motility/ morphology | I | Asthenozoospermia (MMAF) | Lu et al. (2021) | |
| **DNAL1** | Dynein Axonemal Light Chain 1 | Flagellar assembly and motility | I | Asthenozoospermia (MMAF) | Wu et al. (2023) | |
| **DNAH3** | Dynein Axonemal Heavy Chain 3 | Flagellar assembly and motility | I | Asthenozoospermia (MMAF) | Meng et al. (2024) and Wang et al. (2024) | |
| **AKAP3** | A-Kinase Anchoring Protein 3 | Fibrous sheath formation / Sperm motility | I | Asthenozoospermia (MMAF) | Xu et al.(2020) | Liu et al.(2023) |
| **AKAP4** | A-Kinase Anchoring Protein 4 | Fibrous sheath formation / Sperm motility | I | Asthenozoospermia (MMAF) | Miki et al. (2002) | Zhang et al. (2021) |
| **CCDC146** | Coiled-Coil Domain Containing 146 | sperm head and flagellar structure / motility | I | Asthenozoospermia (MMAF) | Ma et al. (2023) | Ye et al. (2024) |
| **CEP135** | Centrosomal Protein 135 | Flagellar formation and function / sperm motility | I | Asthenozoospermia (MMAF) | Liu et al. (2025) | Sha et al.(2017) |
| **CEP78** | Centrosomal Protein 78 | Flagellum biogenesis / sperm motility | I | Asthenozoospermia (MMAF) | Zhang et al.(2022) and Zhu et al.(2023) | |
| **CFAP206** | Cilia And Flagella Associated Protein 206 | Flagellum structure / sperm motility | I | Asthenozoospermia (MMAF) | Shen et al. (2021) | |
| **CFAP47** | Cilia And Flagella Associated Protein 47 | Flagellum structure / sperm motility | I | Asthenozoospermia (MMAF) | Liu et al. (2021) | Liu et al. (2023) |
| **CFAP58** | Cilia And Flagella Associated Protein 58 | Flagellum structure / sperm motility | I | Asthenozoospermia (MMAF) | He et al. (2020) | Sha et al.(2021) |
| **CFAP61** | Cilia And Flagella Associated Protein 61 | Flagellum structure / sperm motility | I | Asthenozoospermia (MMAF) | Huang et al. (2020) | Hu et al. (2023) |
| **DNAH10** | Dynein Axonemal Heavy Chain 10 | Flagellum structure / sperm motility | I | Asthenozoospermia (MMAF) | Tu et al.(2021) | Li et al.(2022) |
| **DNAH8** | Dynein Axonemal Heavy Chain 8 | Flagellum structure / sperm motility | I | Asthenozoospermia (MMAF) | Liu et al. (2020) | |
| **DNHD1** | Dynein Heavy Chain Domain 1 | Flagellum structure / sperm motility | I | Asthenozoospermia (MMAF) | Tan et al.(2022) | Martinez et al. (2023) |
| **DRC1** | Dynein Regulatory Complex Subunit 1 | Flagellum structure / sperm motility | I | Asthenozoospermia (MMAF) | Zhang et al.(2021) | |
| **DZIP1** | DAZ Interacting Zinc Finger Protein 1 | Flagellum structure / sperm motility | I | Asthenozoospermia (MMAF) | Lv et al. (2020) | |
| **IFT74** | Intraflagellar Transport 74 | Flagellum structure / sperm motility | I | Asthenozoospermia (MMAF) | Shi et al. (2019) | Lorès et al. (2021) |
| **NPHP4** | Nephrocystin 4 | Flagellum structure / sperm motility | I | Asthenozoospermia (MMAF) | Won et al. (2011) | Ali et al. (2024) |
| **ODF2** | Outer Dense Fiber Of Sperm Tails 2 | Flagellum structure / sperm motility | I | Asthenozoospermia (MMAF) | Ito et al. (2019) | Zhu et al. (2022) |
| **STK33** | Serine/Threonine Kinase 33 | Flagellum structure / sperm motility | I | Asthenozoospermia (MMAF) | Martins et al.(2018) | Ma et al. (2021) |
| **DNAH1** | Dynein Axonemal Heavy Chain 1 | Flagellum structure / sperm motility | I | Asthenozoospermia (MMAF) | Khan et al. (2023) | Zhuang et al. (2022) |
| **DNAH2** | Dynein Axonemal Heavy Chain 2 | Flagellum structure / sperm motility | I | Asthenozoospermia (MMAF) | Hwang et al. (2021) | Gao et al. (2022) |
| **DNAH17** | Dynein Axonemal Heavy Chain 17 | Flagellum structure / sperm motility | I | Asthenozoospermia (MMAF) | Zhang et al. (2021) | |
| **CFAP43** | Cilia And Flagella Associated Protein 43 | Flagellum structure / sperm motility | I | Asthenozoospermia (MMAF) | Yu et al. (2021) | Tang et al. (2017) |
| **CFAP44** | Cilia And Flagella Associated Protein 44 | Flagellum structure / sperm motility | I | Asthenozoospermia (MMAF) | Muroňová et al.(2025) | Coutton et al. (2018) |
| **CFAP65** | Cilia And Flagella Associated Protein 65 | Acrosome biogenesis / mitochondrial sheath assembly | I | Asthenozoospermia (MMAF) | Wang et al. (2021) | Li et al. (2020) |
| **CFAP69** | Cilia And Flagella Associated Protein 69 | Sperm motility /morphology | I | Asthenozoospermia (MMAF) | He et al. (2019) | |
| **CFAP70** | Cilia And Flagella Associated Protein 70 | Flagellum structure / sperm motility | I | Asthenozoospermia (MMAF) | Chen et al.(2023) | Beurois et al.(2019) |
| **FSIP2** | Fibrous Sheath Interacting Protein 2 | Flagellum biogenesis / head shaping | I | Asthenozoospermia (MMAF) | Fang et al.(2021) | Hou et al.(2022) |
| **ARMC2** | Armadillo Repeat Containing 2 | Flagellum structure | I | Asthenozoospermia (MMAF) | Coutton et al. (2019) | Khan et al. (2021) |
| **QRICH2** | Glutamine Rich 2 | Flagellum biogenesis | I | Asthenozoospermia (MMAF) | Shen et al. (2019) | Kherraf et al. (2019) |
| **TTC21A** | Tetratricopeptide Repeat Domain 21A | Flagellum structure / sperm motility | I | Asthenozoospermia (MMAF) | Liu et al. (2019) | |
| **TTC29** | Tetratricopeptide Repeat Domain 29 | Flagellum structure / sperm motility | I | Asthenozoospermia (MMAF) | Liu et al. (2019) | |
| **SPEF2** | Sperm Flagellar 2 | Flagellum structure / sperm motility | I | Asthenozoospermia (MMAF) | Lehti et al.(2017) | Lu et al.(2024) |
| **SPAG17** | Sperm Associated Antigen 17 | Sperm motility and flagellar structure | I | Asthenozoospermia(MMAF) | Kazarian et al.(2018) | Xu et al. (2017) |
| **SEPT4** | Septin 4 | Midpiece structure and mitochondrial sheath / motility | I | Asthenoteratozoospermia | Kissel et al. (2005) | Wang et al. (2022) |
| **DAZL** | Deleted in Azoospermia-Like | Germ cell differentiation | I | Azoospermia | Rilianawati et al.(2003) | Teng et al. (2012) |
| **PRM1** | Protamine 1 | Histone to protamine exchange | I | Azoospermia (DNA Fragmentation) | Cho et al. (2001) | Ravel et al. (2007) |
| **PRM2** | Protamine 2 | Histone to protamine exchange | I | Azoospermia (DNA Fragmentation) | Cho et al. (2001) | Yang et al. (2016) |
| **LHβ** | beta subunit of luteinizing hormone (LH) | Testosterone production | I | Azoospermia (Hormonal) | Sairam and Krishnamurthy (2001) | Valdes-Socin et al. (2004) |
| **FSHβ** | Follicle-stimulating hormone (FSH) | Spermatogenesis | I | Azoospermia (Hormonal) | Ma et al. (2004) | Lindstedt et al. (1998) |
| **USP26** | Ubiquitin Specific Peptidase 26 | Enzymatic activity | I | Azoospermia (NOA) | Tian et al. (2019) | Ma et al. (2016) |

| | | | | | | |
|---|---|---|---|---|---|---|
| **HORMAD1** | HORMA Domain Containing 1 | Meiosis | I | Azoospermia (NOA) | Shin et al. (2010) | Miyamoto et al. (2012) |
| **SYCP2** | Synaptonemal Complex Protein 2 | Meiosis | I | Azoospermia (NOA) | Yang et al. (2006) | Schilit et al. (2020) |
| **SYCP3** | Synaptonemal Complex Protein 3 | Meiosis | I | Azoospermia (NOA) | Yuan et al. (2000) | Miyamoto et al. (2003) |
| **SYCE1** | Synaptonemal Complex Central Element Protein 1 | Meiosis | I | Azoospermia (NOA) | Bolcun-Filas et al. (2009) | Maor-Sagie et al. (2015) |
| **MEIOB** | Meiosis Specific With OB Domains | Meiosis | I | Azoospermia (NOA) | Luo et al. (2013) | Gershoni et al. (2017) |
| **DMC1** | DNA Meiotic Recombinase 1 | Meiosis | I | Azoospermia (NOA) | Yoshida et al. (1998) | He et al. (2018) |
| **XRCC2** | X-Ray Repair Cross Complementing 2 | Meiosis | I | Azoospermia (NOA) | Yang et al. (2018) | |
| **ME11** | Meiosis Inhibitor 1 | Meiosis | I | Azoospermia (NOA) | Libby et al. (2002) | Ben Khelifa et al. (2018) |
| **TEX11** | Testis Expressed 11 | Meiosis | I | Azoospermia (NOA) | Adelman and Petrini (2008) | Yang et al. (2015) Yatsenko et al. (2015) |
| **TEX15** | Testis Expressed 15 | Meiosis | I | Azoospermia (NOA) | Yang et al. (2008) | Okutman et al. (2015) Colombo et al. (2017) |
| **PSMC3IP (HOP2)** | Proteasome 26S Subunit, ATPase 3 Interacting Protein | Meiosis | I | Azoospermia (NOA) | Petukhova et al. (2003) | Al-Agha et al. (2018) |
| **HFM1** | Helicase Family Member 1 | Meiosis | I | Azoospermia (NOA) | Guiraldelli et al. (2013) | Zhang et al. (2017) |
| **RNF212** | Ring Finger Protein 212 | Meiosis | I | Azoospermia (NOA) | Fujiwara et al. (2015) | Riera-Escamilla et al. (2019) |
| **STAG3** | Stromal Antigen 3 | Meiosis | I | Azoospermia (NOA) | Fukuda et al. (2014) | Riera-Escamilla et al. (2019) |
| **FKBP6** | FK506 Binding Protein 6 | Meiosis | I | Azoospermia (NOA) | Crackower et al. (2003) | Miyamato et al. (2006) |
| **TDRD9** | Tudor Domain Containing 9 | Chromatoid body | I | Azoospermia (NOA) | Shoji et al. (2009) | Arafat et al. (2017) |
| **TDRD6** | Tudor Domain Containing 6 | Chromatoid body | I | Azoospermia (NOA) | Vasileva et al. (2009) | Sha et al. (2018) |
| **TDRD7** | Tudor Domain Containing 7 | Chromatoid body | I | Azoospermia (NOA) | Tanaka et al. (2011) | Tan et al. (2019) |
| **DDX25** | DEAD-Box Helicase 25 | Enzymatic activity | I | Azoospermia (NOA) | Tsai-Morris et al. (2004) | Tsai-Morris et al. (2007) |
| **SOHLH1** | Spermatogenesis and Oogenesis Specific Basic Helix-Loop-Helix 1 | Transcription factor | I | Azoospermia (NOA) | Ballow et al. (2006) | Choi et al. (2010) |
| **WT1** | Wilms Tumor 1 | Transcription factor | I | Azoospermia (NOA) | Wang et al. (2013) | Seabra et al. (2015) |
| **E2F1** | E2F Transcription Factor 1 | Transcription factor | I | Azoospermia (NOA) | Hoja et al. (2004) | Jorgez et al. (2015) |
| **TAF4B** | TATA-Box Binding Protein Associated Factor 4b | Transcription factor | I | Azoospermia (NOA) | Falender et al. (2005) | Ayhan et al. (2014) |
| **ZMYND15** | Zinc Finger MYND-Type Containing 15 | Transcription factor | I | Azoospermia (NOA) | Yan et al. (2010) | Ayhan et al. (2014) |
| **TAF7L** | TATA-Box Binding Protein Associated Factor 7 Like | Transcription factor | I | Azoospermia (NOA) | Cheng et al. (2007) | Akinloye et al. (2007) |
| **SPATA22** | Spermatogenesis Associated 22 | Meiosis | I | Azoospermia (NOA) | La Salle et al. (2012) | Wu et al., (2021) |
| **PRDM9** | PR/SET Domain 9 | Meiosis | I | Azoospermia (NOA) | Brick et al. (2012) | Hayashi et al., (2005) |
| **STX2** | Syntaxin 2 | Meiosis | I | Azoospermia (NOA) | Fujiwara et al. (2013) | Nakamura et al. (2018) |
| **ZSWIM7** | zinc finger SWIM domain-containing protein 7 | Spermatogenesis (Meiotic recombination) | I | Azoospermia (NOA) | Li et al. (2021) | |
| **TNP1** | TNP1 (Transition Protein 1) | Histone to protamine exchange | I | DNA fragmentation (Abnormal Protamination) | Yu et al. (2000) | Miyagawa et al. (2005) |
| **TNP2** | TNP1 (Transition Protein 2) | Histone to protamine exchange | I | DNA fragmentation (Abnormal Protamination) | Zhao et al. (2001) | Miyagawa et al. (2005) |
| **MFSD6L** | Major Facilitator Superfamily Domain Containing 6 Like | Acrosome formation | I | Oligoasthenoteratozoospermia (OAT) | D. Zhou et al. (2024) | |
| **CCDC157** | Coiled-Coil Domain Containing 157 | Acrosome structure and head-tail coupling apparatus | I | Oligoasthenoteratozoospermia (OAT) | Zheng et al. (2024) | |
| **TENT5D** | Terminal Nucleotidyltransferase 5D | RNA stability | I | Oligoasthenoteratozoospermia (OAT) | Sha et al. (2023) | |
| **BRWD1** | Bromodomain And WD Repeat Domain Containing 1 | Spermiogenesis / Chromatin remodeling | I | Oligoasthenoteratozoospermia (OAT) | Philipps et al. (2008) | Guo et al. (2021) |
| **IFT140** | Intraflagellar Transport 140 | Flagellar assembly and motility | I | Oligoasthenoteratozoospermia (OAT) | Zhang et al. (2018) | Wang et al. (2019) |
| **KATNB1** | Katanin Regulatory Subunit B1 | Flagellar assembly and motility / Manchette structure | I | Oligoasthenoteratozoospermia (OAT) | O'Donnell et al. (2012) | O'Donnell et al. (2014) |
| **MKRN2** | Makorin Ring Finger Protein 2 | Flagellar assembly and motility | I | Oligoasthenoteratozoospermia (OAT) | Qian et al. (2016) | |
| **SEPT12** | Septin 12 | Flagellar assembly and motility / Manchette structure | I | Oligoasthenoteratozoospermia (OAT) | Lin et al. (2009) | Kuo et al. (2012) |
| **UBE2B** | Ubiquitin Conjugating Enzyme E2 B | Head morphology | I | Teratozoospermia | Escalier et al. (2003) | Suryavathi et al. (2008) |
| **SUN5** | Sad1 And UNC84 Domain Containing 5 | Head-tail coupling apparatus | I | Teratozoospermia (Acephalic sperm) | Zhu et al. (2016) | Shang et al. (2017) |
| **PMFBP1** | Polyamine Modulated Factor Binding Protein 1 | Head-tail coupling apparatus | I | Teratozoospermia (Acephalic sperm) | Zhu et al. (2018) | |
| **CCDC188** | Coiled-Coil Domain Containing 188 | Head-tail coupling apparatus | I | Teratozoospermia (Acephalic sperm) | Qiu et al. (2025) | Wang et al. (2024) |
| **CFAP52** | Cilia And Flagella Associated Protein 52 | Head-tail coupling apparatus | I | Teratozoospermia (Acephalic sperm) | Jin et al. (2023) | |
| **BRDT** | Bromodomain Testis Associated | Germ cell differentiation | I | Teratozoospermia (Acephalic sperm) | Gaucher et al. (2012) | Li et al. (2017) |
| **TSGA10** | Testis Specific 10 | sperm motility / mitochondrial sheath arrangement (mice) Headless sperm (human) | I | Teratozoospermia (Acephalic sperm) | Luo et al. (2021) | Sha et al. (2018) |
| **HOOK1** | Hook Microtubule-Tethering Protein 1 | Manchette-nucleus connection | I | Teratozoospermia (Acephalic sperm) | Mendoza-Lujambio et al. (2002) | Chen et al. (2018) |
| **CEP112** | Centrosomal Protein 112 | mRNA translation | I | Teratozoospermia (Acephalic sperm) + OAT | Zhang et al. (2024) | |
| **SPACA1** | Sperm Acrosome Associated 1 | Acrosome formation / Sperm-oocyte fusion | I | Teratozoospermia (Globozoospermia) | Fujihara et al. (2012) | Chen et al. (2021) |
| **PICK1** | Protein Interacting With PRKCA 1 | Acrosome formation | I | Teratozoospermia (Globozoospermia) | Xiao et al. (2009) | Liu et al. (2010) |
| **SPATA16** | Spermatogenesis Associated 16 | Human sperm head shape; mouse spermiogenic arrest | I | Teratozoospermia (Globozoospermia) | Fujihara et al. (2017) | Dam et al. (2007) |
| **ZPBP1** | Zona Pellucida Binding Protein 1 | ZP binding to oocyte | I | Teratozoospermia (Globozoospermia) | Lin et al. (2007) | Yatsenko et al. (2012) |
| **DPY19L2** | DPY19 Like 2 | Acrosome integrity | I | Teratozoospermia (Globozoospermia) | Pierre et al. (2012) | Celse et al. (2021) |
| **SPINK2** | Serine Peptidase Inhibitor, Kazal Type 2 | Acrosome formation | I | Teratozoospermia (Globozoospermia) | Kherraf et al. (2017) | |
| **CCDC62** | Coiled-Coil Domain Containing 62 | Acrosome formation | I | Teratozoospermia (Globozoospermia) | Li et al. (2017) | Oud et al. (2020) |
| **AURKC** | Aurora Kinase C | Meiosis | I | Teratozoospermia (Macrozoospermia) | Yang et al. (2010) | Ounis et al. (2015); Hua and Wan (2019). |
| **ACTL7A** | Actin Like 7A | acrosome biogenesis and attachment | I | Total Fertilization Failure (TFF) /OAD | Ferrer et al. (2023) | Xin et al. (2020) |
| **PLCZ1** | Phospholipase C zeta 1 | Calcium oscillations | S | Total Fertilization Failure (TFF) /OAD | Nozawa et al. (2018) | Kashir et al. (2012) |
| **ACTL9** | Actin Like 9 | Perinuclear theca structure | I | Total Fertilization Failure (TFF) /OAD | Dai et al. (2021) | |
| **IQCN** | IQ Motif Containing N | Acrosome integrity | I | Total Fertilization Failure (TFF) /OAD | Sha et al. (2022) | |
| **CALR3** | Calreticulin 3 | Sperm migration and ZP binding | I | Total Fertilization Failure (TFF) /OAD | Ikawa et al. (2011) | Gao et al. (2024) |

**References**


Adelman, C. A., & Petrini, J. H. (2008). ZIP4H (TEX11) deficiency in the mouse impairs meiotic double strand break repair and the regulation of crossing over. PLoS Genetics, 4(3), e1000042. https://doi.org/10.1371/journal.pgen.1000042

Akbari, A., Pipitone, G. B., Anvar, Z., Jaafarinia, M., Ferrari, M., Carrera, P., & Totonchi, M. (2019). ADCY10 frameshift variant leading to severe recessive asthenozoospermia and segregating with absorptive hypercalciuria. Human Reproduction, 34(6), 1155–1164. https://doi.org/10.1093/humrep/dez048

Akinloye, O., Gromoll, J., Callies, C., Nieschlag, E., & Simoni, M. (2007). Mutation analysis of the X-chromosome linked, testis-specific TAF7L gene in spermatogenic failure. Andrologia, 39(5), 190-195. https://doi.org/10.1111/j.1439-0272.2007.00789.x

Al-Agha, A. E., Ahmed, I. A., Nuebel, E., Moriwaki, M., Moore, B., Peacock, K. A., ... & Welt, C. K. (2018). Primary ovarian insufficiency and azoospermia in carriers of a homozygous PSMC3IP stop gain mutation. The Journal of Clinical Endocrinology & Metabolism, 103(2), 555-563.https://doi.org/10.1210/jc.2017-01966

Ali, A., Unar, A., Muhammad, Z., Dil, S., Zhang, B., Sadaf, H., Khan, M., Ali, M., Khan, R., Shah, K. M. B., Ma, A., Jiang, X., Zhang, Y., Zhang, H., & Shi, Q. (2024). A novel NPHP4 homozygous missense variant identified in infertile brothers with multiple morphological abnormalities of the sperm flagella. Journal of

Arafat, M., Har-Vardi, I., Harlev, A., Levitas, E., Zeadna, A., Abofoul-Azab, M., ... & Parvari, R. (2017). Mutation in TDRD9 causes non-obstructive azoospermia in infertile men. Journal of medical genetics, 54(9), 633-639.https://doi.org/10.1136/jmedgenet-2017-104514

Asmarinah, Nuraini, T., Sumarsih, T., Paramita, R., Saleh, M. I., Narita, V., Moeloek, N., Steger, K., Hinsch, K.-D., & Hinsch, E. (2012). Mutations in exons 5, 7 and 8 of the human voltage-dependent anion channel type 3 (VDAC3) gene in sperm with low motility. Andrologia, 44, 46–52. https://doi.org/10.1111/j.1439-

Ayhan, O., Balkan, M., Guven, A., Hazan, R., Atar, M., Tok, A., & Tolun, A. (2014). Truncating mutations in TAF4B and ZMYND15 causing recessive azoospermia. Journal of medical genetics, 51(4), 239-244. https://doi.org/10.1136/jmedgenet-2013-102102

Ballow, D., Meistrich, M. L., Matzuk, M., & Rajkovic, A. (2006). Sohlh1 is essential for spermatogonial differentiation. Developmental biology, 294(1), 161-167.https://doi.org/10.1016/j.ydbio.2006.02.027

Beurois, J., Martinez, G., Cazin, C., Kherraf, Z. E., Amiri-Yekta, A., Thierry-Mieg, N., Bidart, M., Petre, G., Satre, V., Brouillet, S., Touré, A., Arnoult, C., Ray, P. F., & Coutton, C. (2019). CFAP70 mutations lead to male infertility due to severe astheno-teratozoospermia. A case report. Human Reproduction, 34(10),

Brick, K., Smagulova, F., Khil, P., Camerini-Otero, R. D., & Petukhova, G. V. (2012). Genetic recombination is directed away from functional genomic elements in mice. Nature, 485(7400), 642-645. https://doi.org/10.1038/nature11089

Cavarocchi, E., Whitfield, M., Chargui, A., Stouvenel, L., Lorès, P., Coutton, C., Arnoult, C., Santulli, P., Patrat, C., Thierry-Mieg, N., Ray, P. F., Dulioust, E., & Touré, A. (2021). The sodium/proton exchanger SLC9C1 (sNHE) is essential for human sperm motility and fertility. Clinical Genetics, 99(5), 684–693.

Celse, T., Cazin, C., Mietton, F., Martinez, G., Martinez, D., Thierry-Mieg, N., ... & Ray, P. F. (2021). Genetic analyses of a large cohort of infertile patients with globozoospermia, DPY19L2 still the main actor, GGN confirmed as a guest player. Human Genetics, 140, 43-57.https://doi.org/10.1007/s00439-020-02229-0

Chen, J., Wang, Y., Wu, B., Shi, H., & Wang, L. (2023). Experimental and molecular support for Cfap70 as a causative gene of 'multiple morphological abnormalities of the flagella' with male infertility. Biology of Reproduction, 109(4), 450–460. https://doi.org/10.1093/biolre/ioad076

Chen, H., Zhu, Y., Zhu, Z., Zhi, E., Lu, K., Wang, X., ... & Xia, W. (2018). Detection of heterozygous mutation in hook microtubule-tethering protein 1 in three patients with decapitated and decaudated spermatozoa syndrome. Journal of medical genetics, 55(3), 150-157.https://doi.org/10.1136/jmedgenet-2016-

Chen, P., Saiyin, H., Shi, R., Liu, B., Han, X., Gao, Y., Ye, X., Zhang, X., & Sun, Y. (2021). Loss of SPACA1 function causes autosomal recessive globozoospermia by damaging the acrosome-acroplaxome complex. Human reproduction (Oxford, England), 36(9), 2587-2596..

Cheng, Y., Buffone, M. G., Kouadio, M., Goodheart, M., Page, D. C., Gerton, G. L., ... & Wang, P. J. (2007). Abnormal sperm in mice lacking the Taf7l gene. Molecular and cellular biology, 27(7), 2582-2589. https://doi.org/10.1128/MCB.01722-06

Cho, C., Willis, W. D., Goulding, E. H., Jung-Ha, H., Choi, Y. C., Hecht, N. B., & Eddy, E. M. (2001). Haploinsufficiency of protamine-1 or -2 causes infertility in mice. Nature genetics, 28(1), 82-86. https://doi.org/10.1038/ng0501-82

Choi, Y., Jeon, S., Choi, M., Lee, M. H., Park, M., Lee, D. R., ... & Shim, S. H. (2010). Mutations in SOHLH1 gene associate with nonobstructive azoospermia. Human mutation, 31(7), 788-793.https://doi.org/10.1002/humu.21264

Colombo, R., Pontoglio, A., & Bini, M. (2017). Two novel TEX15 mutations in a family with nonobstructive azoospermia. Gynecologic and obstetric investigation, 82(3), 283-286.https://doi.org/10.1159/000468934

Coutton, C., Vargas, A. S., Amiri-Yekta, A., Kherraf, Z. E., Ben Mustapha, S. F., Le Tanno, P., Wambergue-Legrand, C., Karaouzène, T., Martinez, G., Crouzy, S., Daneshipour, A., Hosseini, S. H., Mitchell, V., Halouani, L., Marrakchi, O., Makni, M., Latrous, H., Kharouf, M., ... Ray, P. F. (2018). Mutations in CFAP43

Coutton, C., Martinez, G., Kherraf, Z. E., Amiri-Yekta, A., Boguenet, M., Saut, A., He, X., Zhang, F., Cristou-Kent, M., Escoffier, J., Bidart, M., Satre, V., Conne, B., Fourati Ben Mustapha, S., Halouani, L., Marrakchi, O., Makni, M., Latrous, H., ... Ray, P. F. (2019). Bi-allelic mutations in ARMC2 lead to severe astheno-

Crackower, M. A., Kolas, N. K., Noguchi, J., Sarao, R., Kikuchi, K., Kaneko, H., ... & Penninger, J. M. (2003). Essential role of Fkbp6 in male fertility and homologous chromosome pairing in meiosis. Science, 300(5623), 1291-1295.https://doi.org/10.1126/science.1083022

Dai, J., Zhang, T., Guo, J., Zhou, Q., Gu, Y., Zhang, J., Hu, L., Zong, Y., Song, J., Zhang, S., Dai, C., Gong, F., Lu, G., Zheng, W., & Lin, G. (2021). Homozygous pathogenic variants in ACTL9 cause fertilization failure and male infertility in humans and mice. American journal of human genetics, 108(3), 469-481.

Dam AH, Koscinski I, Kremer JA, Moutou C, Jaeger AS, Oudakker AR, Tournaye H, Charlet N, Lagier-Tourenne C, van Bokhoven H et al. Homozygous mutation in SPATA16 is associated with male infertility in human globozoospermia. Am J Hum Genet 2007;81:813-820.https://doi.org/10.1086/521314

Danshina, P. V., Geyer, C. B., Dai, Q., Goulding, E. H., Willis, W. D., Kitto, G. B., McCarrey, J. R., Eddy, E. M., & O'Brien, D. A. (2010). Phosphoglycerate kinase 2 (PGK2) is essential for sperm function and male fertility in mice. Biology of Reproduction, 82(1), 136–145. https://doi.org/10.1095/biolreprod.109.079699

Dirami, T., Rode, B., Jollivet, M., Da Silva, N., Escalier, D., Gaitch, N., Norez, C., Tuffery, P., Wolf, J. P., Becq, F., Ray, P. F., Dulioust, E., Gacon, G., Bienvenu, T., & Touré, A. (2013). Missense mutations in SLC26A8, encoding a sperm-specific activator of CFTR, are associated with human asthenozoospermia.

El Khouri, E., Whitfield, M., Stouvenel, L., Kini, A., Riederer, B., Lores, P., Roemermann, D., di Stefano, G., Drevet, J. R., Saez, F., Seidler, U., & Touré, A. (2018). Slc26a3 deficiency is associated with epididymis dysplasia and impaired sperm fertilization potential in the mouse. Molecular Reproduction and Development,

Elkina, Y. L., Kuravsky, M. L., Bragina, E. E., Kurilo, L. F., Khayat, S. S., Sukhomlinova, M. Y., & Schmalhausen, E. V. (2017). Detection of a mutation in the intron of sperm-specific glyceraldehyde-3-phosphate dehydrogenase gene in patients with fibrous sheath dysplasia of the sperm flagellum. Andrologia,

Escalier D, Bai XY, Silvius D, Xu PX, Xu X. Spermatid nuclear and sperm periaxonemal anomalies in the mouse Ube2b null mutant. Molecular Reproduction and Development: Incorporating Gamete Research. 2003; 65: 298-308. https://doi.org/10.1002/mrd.10290

Esposito, G., Jaiswal, B. S., Xie, F., Krajnc-Franken, M. A., Robben, T. J., Strik, A. M., et al. (2004). Mice deficient for soluble adenylyl cyclase are infertile because of a severe sperm-motility defect. Proceedings of the National Academy of Sciences, 101(8), 2993–2998. https://doi.org/10.1073/pnas.0400050101

Falender, A. E., Freiman, R. N., Geles, K. G., Lo, K. C., Hwang, K., Lamb, D. J., ... & Richards, J. S. (2005). Maintenance of spermatogenesis requires TAF4b, a gonad-specific subunit of TFIID. Genes & development, 19(7), 794-803. https://doi.org/10.1101/gad.1290105

Fang, X., Gamallat, Y., Chen, Z., Mai, H., Zhou, P., Sun, C., Li, X., Li, H., Zheng, S., Liao, C., Yang, M., Li, Y., Yang, Z., Ma, C., Han, D., Zuo, L., Xu, W., Hu, H., Sun, L., & Li, N. (2021). Hypomorphic and hypermorphic mouse models of Fsip2 indicate its dosage-dependent roles in sperm tail and acrosome

Ferrer, P., Upadhyay, S., Ikawa, M., & Clement, T. M. (2023). Testis-specific actin-like 7A (ACTL7A) is an indispensable protein for subacrosomal-associated F-actin formation, acrosomal anchoring, and male fertility. Molecular Human Reproduction, 29(3), gaad005. https://doi.org/10.1093/molehr/gaad005

Fujihara, Y., Satouh, Y., Inoue, N., Isotani, A., Ikawa, M., & Okabe, M. (2012). SPACA1-deficient male mice are infertile with abnormally shaped sperm heads reminiscent of globozoospermia. Development, 139(19), 3583-3589. https://doi.org/10.1242/dev.081778

Fujihara, Y., Oji, A., Larasati, T., Kojima-Kita, K., & Ikawa, M. (2017). Human Globozoospermia-Related Gene Spata16 Is Required for Sperm Formation Revealed by CRISPR/Cas9-Mediated Mouse Models. International journal of molecular sciences, 18(10), 2208. https://doi.org/10.3390/ijms18102208.

| |
|---|
| Fujiwara Y, Matsumoto H, Akiyama K, Srivastava A, Chikushi M, Ann Handel M, Kunieda T. An ENU-induced mutation in the mouse Rnf212 gene is associated with male meiotic failure and infertility. Reproduction 2015;149:67-74.https://doi.org/10.1530/REP-14-0122 |
| Fujiwara Y, Ogonuki N, Inoue K, Ogura A, Handel MA, Noguchi J, Kunieda T. t-SNARE Syntaxin2 (STX2) is implicated in intracellular transport of sulfoglycolipids during meiotic prophase in mouse spermatogenesis. Biol Reprod 2013;88:141.https://doi.org/10.1095/biolreprod.112.107110 |
| Fukuda, T., Fukuda, N., Agostinho, A., Hernández-Hernández, A., Kouznetsova, A., & Höög, C. (2014). STAG 3-mediated stabilization of REC 8 cohesin complexes promotes chromosome synapsis during meiosis. The EMBO journal, 33(11), 1243-1255.https://doi.org/10.1002/embj.201387329 |
| Gao, Y., Tian, S., Sha, Y., Zha, X., Cheng, H., Wang, A., Liu, C., Lv, M., Ni, X., Li, Q., Wu, H., Tan, Q., Tang, D., Song, B., Ding, D., Cong, J., Xu, Y., Zhou, P., Wei, Z., ... He, X. (2021). Novel bi-allelic variants in DNAH2 cause severe asthenoteratozoospermia with multiple morphological abnormalities of the flagella. |
| Gao, Y., Xue, R., Guo, R., Yang, F., Sha, X., Li, Y., Hua, R., Li, G., Shen, Q., Li, K., Liu, W., Xu, Y., Zhou, P., Wei, Z., Zhang, Z., Cao, Y., He, X., & Wu, H. (2024). CALR3 defects disrupt sperm-zona pellucida binding in humans: new insights into male factor fertilization failure and relevant clinical therapeutic |
| Gaucher J, Boussouar F, Montellier E, Curtet S, Buchou T, Bertrand S, Hery P, Jounier S, Depaux A, Vitte AL, Guardiola P, Pernet K, Debernardi A, Lopez F, Holota H, Imbert J, Wolgemuth DJ, Gérard M, Rousseaux S & Khochbin S. (2012) Bromodomain-dependent stage-specific male genome programming by Brdt. EMBO |
| Guiraldelli, M. F., Eyster, C., Wilkerson, J. L., Dresser, M. E., & Pezza, R. J. (2013). Mouse HFM1/Mer3 is required for crossover formation and complete synapsis of homologous chromosomes during meiosis. PLoS genetics, 9(3), e1003383.https://doi.org/10.1371/journal.pgen.1003383 |
| Guo T, Tu C-F, Yang D-H, Ding S-Z, Lei C, Wang R-C, et al. Bi-allelic BRWD1 variants cause male infertility with asthenoteratozoospermia and likely primary ciliary dyskinesia. Human Genetics. 2021: 1-13. https://doi.org/10.1007/s00439-020-02241-4 |
| Hagiuda, J., Takasaki, N., Oya, M., Ishikawa, H., & Narimatsu, H. (2020). Mutation of GALNTL5 gene identified in patients diagnosed with asthenozoospermia. Human Fertility, 23(4), 226–233. https://doi.org/10.1080/14647273.2018.1562239 |
| Harris, T. P., Schimenti, K. J., Munroe, R. J., & Schimenti, J. C. (2014). IQ motif-containing G (Iqcg) is required for mouse spermiogenesis. G3: Genes, Genomes, Genetics, 4(2), 367–372. https://doi.org/10.1534/g3.113.009563 |
| Harris, T., Marquez, B., Suarez, S., & Schimenti, J. (2007). Sperm motility defects and infertility in male mice with a mutation in Nsun7, a member of the Sun domain-containing family of putative RNA methyltransferases. Biology of Reproduction, 77(2), 376-382.https://doi.org/10.1095/biolreprod.106.058669 |
| Hayashi, K., Yoshida, K., & Matsui, Y. (2005). A histone H3 methyltransferase controls epigenetic events required for meiotic prophase. Nature, 438(7066), 374-378.https://doi.org/10.1038/nature04112 |
| He, W. B., Xiao, W. J., Tan, Y. Q., Zhao, X. M., Li, W., Zhang, Q. J., Zhong, C. G., Li, X. R., Hu, L., Lu, G. X., Lin, G., & Du, J. (2018). Novel mutations of PKD genes in Chinese patients suffering from autosomal dominant polycystic kidney disease and seeking assisted reproduction. BMC Medical Genetics, 19(1), 186. |
| He, X., Liu, C., Yang, X., Lv, M., Ni, X., Li, Q., Cheng, H., Liu, W., Tian, S., Wu, H., Gao, Y., Yang, C., Tan, Q., Cong, J., Tang, D., Zhang, J., Song, B., Zhong, Y., Li, H., Zhi, W., Mao, X., Fu, F., Ge, L., Shen, Q., Zhang, M., Saiyin, H., Jin, L., Xu, Y., Zhou, P., Wei, Z., Zhang, F., & Cao, Y. (2020). Bi-allelic loss-of- |
| He, X., Li, W., Wu, H., Lv, M., Liu, W., Liu, C., Zhu, F., Li, C., Fang, Y., Yang, C., Cheng, H., Zhang, J., Tan, J., Chen, T., Tang, D., Song, B., Wang, X., Zha, X., Wang, H., ... Cao, Y. (2019). Novel homozygous CFAP69 mutations in humans and mice cause severe asthenoteratospermia with multiple morphological |
| Hoja, M. R., Liu, J. G., Mohammadieh, M., Kvist, U., & Yuan, L. (2004). E2F1 deficiency impairs murine spermatogenesis and augments testicular degeneration in SCP3-nullizygous mice. Cell Death & Differentiation, 11(3), 354-356.https://doi.org/10.1038/sj.cdd.4401362 |
| Hou, M., Xi, Q., Zhu, L., Jia, W., Liu, Z., Wang, C., Zhou, X., Zhang, D., Xing, C., Peng, X., Luo, Y., Jin, L., Li, Z., & Zhang, X. (2022). Novel compound heterozygous mutation in FSIP2 causes multiple morphological abnormalities of the sperm flagella (MMAF) and male infertility. Reproductive Sciences, 29(9), 2697–2702. |
| Hu, T., Meng, L., Tan, C., Luo, C., He, W. B., Tu, C., Zhang, H., Du, J., Nie, H., Lu, G. X., Lin, G., & Tan, Y. Q. (2023). Biallelic CFAP61 variants cause male infertility in humans and mice with severe oligoasthenoteratozoospermia. Journal of Medical Genetics, 60(2), 144–153. |
| Hua, J., & Wan, Y. Y. (2019). Whole-exome sequencing identified a novel mutation of AURKC in a Chinese family with macrozoospermia. Journal of assisted reproduction and genetics, 36, 529-534. https://doi.org/10.1007/s10815-018-1374-3 |

Huang, T., Yin, Y., Liu, C., Li, M., Yu, X., Wang, X., Zhang, H., Muhammad, T., Gao, F., Li, W., Chen, Z. J., Liu, H., & Ma, J. (2020). Absence of murine CFAP61 causes male infertility due to multiple morphological abnormalities of the flagella. Science Bulletin (Beijing), 65(10), 854–864.

Hwang, J. Y., Nawaz, S., Choi, J., Wang, H., Hussain, S., Nawaz, M., Lopez-Giraldez, F., Jeong, K., Dong, W., Oh, J. N., Bilguvar, K., Mane, S., Lee, C. K., Bystroff, C., Lifton, R. P., Ahmad, W., & Chung, J. J. (2021). Genetic defects in DNAH2 underlie male infertility with multiple morphological abnormalities of the

Ikawa, M., Tokuhiro, K., Yamaguchi, R., Benham, A. M., Tamura, T., Wada, I., Satouh, Y., Inoue, N., & Okabe, M. (2011). Calsperin is a testis-specific chaperone required for sperm fertility. The Journal of biological chemistry, 286(7), 5639-5646.https://doi.org/10.1074/jbc.M110.140152

Ito, C., Akutsu, H., Yao, R., Yoshida, K., Yamatoya, K., Mutoh, T., Makino, T., Aoyama, K., Ishikawa, H., Kunimoto, K., Tsukita, S., Noda, T., Kikkawa, M., & Toshimori, K. (2019). Odf2 haploinsufficiency causes a new type of decapitated and decaudated spermatozoa, Odf2-DDS, in mice. Scientific Reports, 9, 14249.

Jin, H. J., Ruan, T., Dai, S., Geng, X. Y., Yang, Y., Shen, Y., & Chen, S. R. (2023). Identification of CFAP52 as a novel diagnostic target of male infertility with defects of sperm head-tail connection and flagella development. eLife, 12, RP92769. https://doi.org/10.7554/eLife.92769

Jorgez, C. J., Wilken, N., Addai, J. B., Newberg, J., Vangapandu, H. V., Pastuszak, A. W., ... & Lamb, D. J. (2015). Genomic and genetic variation in E2F transcription factor-1 in men with nonobstructive azoospermia. Fertility and sterility, 103(1), 44-52.https://doi.org/10.1016/j.fertnstert.2014.09.021

Kashir, J., Konstantinidis, M., Jones, C., Lemmon, B., Lee, H. C., Hamer, R., Heindryckx, B., Deane, C. M., De Sutter, P., Fissore, R. A., Parrington, J., Wells, D., & Coward, K. (2012). A maternally inherited autosomal point mutation in human phospholipase C zeta (PLCζ) leads to male infertility. Human reproduction (Oxford,

Kazarian, E., Son, H., Sapao, P., Li, W., Zhang, Z., Strauss, J. F., & Teves, M. E. (2018). SPAG17 is required for male germ cell differentiation and fertility. International Journal of Molecular Sciences, 19(4), 1252. https://doi.org/10.3390/ijms19041252

Khan, R., Zaman, Q., Chen, J., Khan, M., Ma, A., Zhou, J., Zhang, B., Ali, A., Naeem, M., Zubair, M., Zhao, D., Shah, W., Khan, M., Zhang, Y., Xu, B., Zhang, H., & Shi, Q. (2021). Novel loss-of-function mutations in DNAH1 displayed different phenotypic spectrum in humans and mice. Frontiers in Endocrinology, 12,

Khan, I., Dil, S., Zhang, H., Zhang, B., Khan, T., Zeb, A., Zhou, J., Nawaz, S., Zubair, M., Khan, K., Ma, H., & Shi, Q. (2021). A novel stop-gain mutation in ARMC2 is associated with multiple morphological abnormalities of the sperm flagella. Reproductive Biomedicine Online, 43(5), 913–919.

Kherraf, Z. E., Barbotin, A. L., Martinez, G., Mazet, A., Cazin, C., Coutton, C., Arnoult, C., Thierry-Mieg, N., Rives, N., Rives-Feraille, A., & Ray, P. F. (2024). A splice donor variant of GAS8 induces structural disorganization of the axoneme in sperm flagella and leads to nonsyndromic male infertility. Clinical

Kherraf, Z. E., Cazin, C., Coutton, C., Amiri-Yekta, A., Martinez, G., Boguenet, M., ... (2019). Whole exome sequencing of men with multiple morphological abnormalities of the sperm flagella reveals novel homozygous QRICH2 mutations. Clinical Genetics, 96, 394–401. https://doi.org/10.1111/cge.13604

Kherraf ZE, Christou-Kent M, Karaouzene T, Amiri-Yekta A, Martinez G, Vargas AS, Lambert E, Borel C, Dorphin B, AkninSeifer I et al. SPINK2 deficiency causes infertility by inducing sperm defects in heterozygotes and azoospermia in homozygotes. EMBO Mol Med 2017;9:1132-1149.

Khosronezhad, N., Hosseinzadeh Colagar, A., & Mortazavi, S. M. (2015). The Nsun7 (A11337)-deletion mutation, causes reduction of its protein rate and associated with sperm motility defect in infertile men. Journal of assisted reproduction and genetics, 32, 807-815.https://doi.org/10.1007/s10815-015-0443-0

Kissel H, Georgescu M-M, Larisch S, Manova K, Hunnicutt GR, Steller H. The Sept4 septin locus is required for sperm terminal differentiation in mice. Developmental cell. 2005; 8: 353-64.https://doi.org/10.1016/j.devcel.2005.01.021

Kuo YC, Lin YH, Chen HI, Wang YY, Chiou YW, Lin HH, et al. SEPT12 mutations cause male infertility with defective sperm annulus. Human mutation. 2012; 33: 710-9.https://doi.org/10.1002/humu.22028

La Salle, S., Palmer, K., O'Brien, M., Schimenti, J. C., Eppig, J., & Handel, M. A. (2012). Spata22, a novel vertebrate-specific gene, is required for meiotic progress in mouse germ cells. Biology of Reproduction, 86(2), 45. https://doi.org/10.1095/biolreprod.111.095752

Lehti, M. S., Zhang, F. P., Kotaja, N., & Sironen, A. (2017). SPEF2 functions in microtubule-mediated transport in elongating spermatids to ensure proper male germ cell differentiation. Development, 144(14), 2683–2693. https://doi.org/10.1242/dev.152108

Li, R. K., Tan, J. L., Chen, L. T., Feng, J. S., Liang, W. X., Guo, X. J., Liu, P., Chen, Z., Sha, J. H., Wang, Y. F., & Chen, S. J. (2014). Iqcg is essential for sperm flagellum formation in mice. PLoS ONE, 9(5), e98053. https://doi.org/10.1371/journal.pone.0098053

| |
|---|
| Li, K., Wang, G., Lv, M., Wang, J., Gao, Y., Tang, F., Xu, C., Yang, W., Yu, H., Shao, Z., Geng, H., Tan, Q., Shen, Q., Tang, D., Ni, X., Wang, T., Song, B., Wu, H., Huo, R., ... Cao, Y. (2022). Bi-allelic variants in DNAH10 cause asthenoteratozoospermia and male infertility. Journal of Assisted Reproduction and Genetics, |
| Li, W., Wu, H., Li, F., Tian, S., Kherraf, Z. E., Zhang, J., Ni, X., Lv, M., Liu, C., Tan, Q., Shen, Y., Amiri-Yekta, A., Cazin, C., Zhang, J., Liu, W., Zheng, Y., Cheng, H., Wu, Y., Wang, J., ... Zhang, F. (2020). Biallelic mutations in CFAP65 cause male infertility with multiple morphological abnormalities of the sperm |
| Li, L., Sha, Y., Wang, X., Li, P., Wang, J., Kee, K., & Wang, B. (2017). Whole-exome sequencing identified a homozygous BRDT mutation in a patient with acephalic spermatozoa. Oncotarget, 8(12), 19914-19922. https://doi.org/10.18632/oncotarget.15251 |
| Li, Y., Wu, Y., Zhou, J., Zhang, H., Zhang, Y., Ma, H., Jiang, X., & Shi, Q. (2021). A recurrent ZSWIM7 mutation causes male infertility resulting from decreased meiotic recombination. Human reproduction (Oxford, England), 36(5), 1436-1445.https://doi.org/10.1093/humrep/deab046 |
| Lin, Y. N., Roy, A., Yan, W., Burns, K. H., & Matzuk, M. M. (2007). Loss of zona pellucida binding proteins in the acrosomal matrix disrupts acrosome biogenesis and sperm morphogenesis. Molecular and cellular biology, 27(19), 6794-6805.https://doi.org/10.1128/MCB.01029-07 |
| Lindstedt, G., Nyström, E., Matthews, C., Ernest, I., Janson, P. O., & Chatterjee, K. (1998). Follitropin (FSH) deficiency in an infertile male due to FSHbeta gene mutation. A syndrome of normal puberty and virilization but underdeveloped testicles with azoospermia, low FSH but high lutropin and normal serum testosterone |
| Lin Y-H, Lin Y-M, Wang Y-Y, Yu I-S, Lin Y-W, Wang Y-H, et al. The expression level of septin12 is critical for spermiogenesis. The American journal of pathology. 2009; 174: 1857-68.https://doi.org/10.2353/ajpath.2009.080955 |
| Liu, Y., Li, Y., Meng, L., Li, K., Gao, Y., Lv, M., Guo, R., Xu, Y., Zhou, P., Wei, Z., He, X., Cao, Y., Wu, H., Tan, Y., & Hua, R. (2023). Bi-allelic human TEKT3 mutations cause male infertility with oligoasthenoteratozoospermia owing to acrosomal hypoplasia and reduced progressive motility. Human |
| Liu, X. X., Zhang, H., Shen, X. F., Liu, F. J., Liu, J., & Wang, W. J. (2016). Characteristics of testis-specific phosphoglycerate kinase 2 and its association with human sperm quality. Human Reproduction, 31(2), 273–279. https://doi.org/10.1093/humrep/dev301 |
| Liu, C., Shen, Y., Tang, S., Wang, J., Zhou, Y., Tian, S., Wu, H., Cong, J., He, X., Jin, L., Cao, Y., Yang, Y., & Zhang, F. (2023). Homozygous variants in AKAP3 induce asthenoteratozoospermia and male infertility. Journal of Medical Genetics, 60(2), 137–143. https://doi.org/10.1136/jmedgenet-2021-108271 |
| Liu, H., Zhang, H., Qin, G., Song, T., Liu, X., Wen, Z., Liu, M., Wang, X., Fu, X., & Gao, J. (2025). Loss of Cep135 causes oligoasthenoteratozoospermia and male infertility in mice. Cellular and Molecular Life Sciences, 82(1), 117. https://doi.org/10.1007/s00018-025-05616-w |
| Liu, C., Tu, C., Wang, L., Wu, H., Houston, B. J., Mastrorosa, F. K., Zhang, W., Shen, Y., Wang, J., Tian, S., Meng, L., Cong, J., Yang, S., Jiang, Y., Tang, S., Zeng, Y., Lv, M., Lin, G., Li, J., Saiyin, H., He, X., Jin, L., Touré, A., Ray, P. F., Veltman, J. A., Shi, Q., O'Bryan, M. K., Cao, Y., Tan, Y. Q., & Zhang, F. (2021). |
| Liu, M., Dai, S., Zhang, J., Yang, Y., Shen, Y., Liu, H., Yang, Y., Jiang, C., & Tian, E. (2023). A novel mutation in CFAP47 causes male infertility due to multiple morphological abnormalities of the sperm flagella. Frontiers in Endocrinology, 14, 1155639. https://doi.org/10.3389/fendo.2023.1155639 |
| Liu, C., Miyata, H., Gao, Y., Sha, Y., Tang, S., Xu, Z., Whitfield, M., Patrat, C., Wu, H., Dulioust, E., Tian, S., Shimada, K., Cong, J., Noda, T., Li, H., Morohoshi, A., Cazin, C., Kherraf, Z. E., Arnoult, C., ... Zhang, F. (2020). Bi-allelic DNAH8 variants lead to multiple morphological abnormalities of the sperm flagella and |
| Liu, W., He, X., Yang, S., Zouari, R., Wang, J., Wu, H., Kherraf, Z. E., Liu, C., Coutton, C., Zhao, R., Tang, D., Tang, S., Lv, M., Fang, Y., Li, W., Li, H., Zhao, J., Wang, X., Zhao, S., ... Zhang, F. (2019). Bi-allelic mutations in TTC21A induce asthenoteratospermia in humans and mice. American Journal of Human |
| Liu, C., He, X., Liu, W., Yang, S., Wang, L., Li, W., Wu, H., Tang, S., Ni, X., Wang, J., Gao, Y., Tian, S., Zhang, L., Cong, J., Zhang, Z., Tan, Q., Zhang, J., Li, H., Zhong, Y., ... Zhang, F. (2019). Bi-allelic mutations in TTC29 cause male subfertility with asthenoteratospermia in humans and mice. American Journal of Human |
| Liu G, Shi Q-W, Lu G-X. A newly discovered mutation in PICK1 in a human with globozoospermia. Asian journal of andrology. 2010; 12: 556. https://doi.org/10.1038/aja.2010.47 |
| Li Y, Li C, Lin S, Yang B, Huang W, Wu H, et al. A nonsense mutation in Ccdc62 gene is responsible for spermiogenesis defects and male infertility in repro29/repro29 mice. Biology of Reproduction. 2017; 96:587-97. https://doi.org/10.1095/biolreprod.116.141408 |
| Lorès, P., Kherraf, Z. E., Amiri-Yekta, A., Whitfield, M., Daneshipour, A., Stouvenel, L., Cazin, C., Cavarocchi, E., Coutton, C., Llabador, M. A., Arnoult, C., Thierry-Mieg, N., Ferreux, L., Patrat, C., Hosseini, S. H., Mustapha, S. F. B., Zouari, R., Dulioust, E., Ray, P. F., & Touré, A. (2021). A missense mutation in |

| |
|---|
| Lu, S., Gu, Y., Wu, Y., Yang, S., Li, C., Meng, L., Yuan, W., Jiang, T., Zhang, X., Li, Y., Wang, C., Liu, M., Ye, L., Guo, X., Shen, H., Yang, X., & Hu, Z. (2021). Bi-allelic variants in human WDR63 cause male infertility via abnormal inner dynein arms assembly. Cell Discovery, 7(1), 110. https://doi.org/10.1038/s41421- |
| Lu, W., Li, Y., Meng, L., Tan, C., Nie, H., Zhang, Q., Song, Y., Zhang, H., Tan, Y. Q., Tu, C., Guo, H., Wu, L., & Du, J. (2024). Novel SPEF2 variants cause male infertility and likely primary ciliary dyskinesia. Journal of Assisted Reproduction and Genetics, 41(6), 1485–1498. https://doi.org/10.1007/s10815-024-03106-9 |
| Luo, G., Hou, M., Wang, B., Liu, Z., Liu, W., Han, T., Zhang, D., Zhou, X., Jia, W., Tan, Y., Wu, Y., Wang, J., & Zhang, X. (2021). Tsga10 is essential for arrangement of mitochondrial sheath and male fertility in mice. Andrology, 9(1), 368-375. https://doi.org/10.1111/andr.12889 |
| Lv, M., Liu, C., Ma, C., Yu, H., Shao, Z., Gao, Y., Liu, Y., Wu, H., Tang, D., Tan, Q., Zhang, J., Li, K., Xu, C., Geng, H., Zhang, J., Li, H., Mao, X., Ge, L., Fu, F., Zhong, K., Xu, Y., Tao, F., Zhou, P., Wei, Z., He, X., Zhang, F., & Cao, Y. (2022). Homozygous mutation in SLO3 leads to severe asthenoteratozoospermia due to |
| Lv, M., Liu, W., Chi, W., Ni, X., Wang, J., Cheng, H., Li, W. Y., Yang, S., Wu, H., Zhang, J., Gao, Y., Liu, C., Li, C., Yang, C., Tan, Q., Tang, D., Zhang, J., Song, B., Chen, Y. J., ... Cao, Y. (2020). Homozygous mutations in DZIP1 can induce asthenoteratospermia with severe MMAF. Journal of Medical Genetics, 57(7), |
| Ma, Y., Wu, B., Chen, Y., Ma, S., Wang, L., Han, T., Lin, X., Yang, F., Liu, C., Zhao, J., & Li, W. (2023). CCDC146 is required for sperm flagellum biogenesis and male fertility in mice. Cellular and Molecular Life Sciences, 81(1), 1. https://doi.org/10.1007/s00018-023-05025-x |
| Ma, H., Zhang, B., Khan, A., Zhao, D., Ma, A., Zhou, J., Khan, I., Khan, K., Zhang, H., Zhang, Y., Jiang, X., Dil, S., Zeb, A., Rahim, F., & Shi, Q. (2021). Novel frameshift mutation in STK33 is associated with asthenozoospermia and multiple morphological abnormalities of the flagella. Human Molecular Genetics, |
| Ma, X., Dong, Y., Matzuk, M. M., & Kumar, T. R. (2004). Targeted disruption of luteinizing hormone beta-subunit leads to hypogonadism, defects in gonadal steroidogenesis, and infertility. Proceedings of the National Academy of Sciences of the United States of America, 101(49), 17294-17299. |
| Ma, Q., Li, Y., Guo, H., Li, C., Chen, J., Luo, M., ... & Gui, Y. (2016). A novel missense mutation in USP26 gene is associated with nonobstructive azoospermia. Reproductive Sciences, 23(10), 1434-1441. https://doi.org/10.1177/1933719116641758 |
| Martinez, G., Barbotin, A. L., Cazin, C., Wehbe, Z., Boursier, A., Amiri-Yekta, A., Daneshipour, A., Hosseini, S. H., Rives, N., Feraille, A., Thierry-Mieg, N., Bidart, M., Satre, V., Arnoult, C., Ray, P. F., Kherraf, Z. E., & Coutton, C. (2023). New mutations in DNHD1 cause multiple morphological abnormalities of the sperm |
| Martinez, G., Barbotin, A. L., Cazin, C., Wehbe, Z., Boursier, A., Amiri-Yekta, A., Daneshipour, A., Hosseini, S. H., Rives, N., Feraille, A., Thierry-Mieg, N., Bidart, M., Satre, V., Arnoult, C., Ray, P. F., Kherraf, Z. E., & Coutton, C. (2023). New mutations in DNHD1 cause multiple morphological abnormalities of the sperm |
| Martins, L. R., Bung, R. K., Koch, S., Richter, K., Schwarzmüller, L., Terhardt, D., Kurtulmus, B., Niehrs, C., Rouhi, A., Lohmann, I., Pereira, G., Fröhling, S., Glimm, H., & Scholl, C. (2018). Stk33 is required for spermatid differentiation and male fertility in mice. Developmental Biology, 433(1), 84–93. |
| Mendoza-Lujambio I, Burfeind P, Dixkens C, Meinhardt A, HoyerFender S, Engel W, Neesen J. The Hook1 gene is non-functional in the abnormal spermatozoon head shape (AZH) mutant mouse. Hum Mol Genet 2002;11:1647-1658. https://doi.org/10.1093/hmg/11.14.1647 |
| Meng, Z., Meng, Q., Gao, T., Zhou, H., Xue, J., Li, H., Wu, Y., & Lv, J. (2023). Identification of bi-allelic KIF9 loss-of-function variants contributing to asthenospermia and male infertility in two Chinese families. Frontiers in Endocrinology, 13, 1091107. https://doi.org/10.3389/fendo.2022.1091107 |
| Meng, G. Q., Wang, Y., Luo, C., Tan, Y. M., Li, Y., Tan, C., Tu, C., Zhang, Q. J., Hu, L., Zhang, H., Meng, L. L., Liu, C. Y., Deng, L., Lu, G. X., Lin, G., Du, J., Tan, Y. Q., Sha, Y., & Wang, L., He, W. B. (2024). Bi-allelic variants in DNAH3 cause male infertility with asthenoteratozoospermia in humans and mice. Human |
| Miki, K., Qu, W., Goulding, E. H., Willis, W. D., Bunch, D. O., Strader, L. F., Perreault, S. D., Eddy, E. M., & O'Brien, D. A. (2004). Glyceraldehyde 3-phosphate dehydrogenase-S, a sperm-specific glycolytic enzyme, is required for sperm motility and male fertility. Proceedings of the National Academy of Sciences, 101(47), |
| Miki, K., Willis, W. D., Brown, P. R., Goulding, E. H., Fulcher, K. D., & Eddy, E. M. (2002). Targeted disruption of the Akap4 gene causes defects in sperm flagellum and motility. Developmental Biology, 248(2), 331–342. https://doi.org/10.1006/dbio.2002.0728 |
| Miyamato, T., Sato, H., Yogev, L., Kleiman, S., Namiki, M., Koh, E., Sakugawa, N., Hayashi, H., Ishikawa, M., Lamb, D. J., & Sengoku, K. (2006). Is a genetic defect in Fkbp6 a common cause of azoospermia in humans?. Cellular & molecular biology letters, 11(4), 557-569.https://doi.org/10.2478/s11658-006-0043-1 |
| Miyamoto, T., Tsujimura, A., Miyagawa, Y., Koh, E., Namiki, M., Horikawa, M., Saijo, Y., & Sengoku, K. (2012). Single-nucleotide polymorphisms in HORMAD1 may be a risk factor for azoospermia caused by meiotic arrest in Japanese patients. Asian journal of andrology, 14(4), 580-583. |

| |
|---|
| Miyata, H., Shimada, K., Morohoshi, A., Oura, S., Matsumura, T., Xu, Z., Oyama, Y., & Ikawa, M. (2020). Testis-enriched kinesin KIF9 is important for progressive motility in mouse spermatozoa. FASEB Journal, 34(4), 5389–5400. https://doi.org/10.1096/fj.201902755R |
| Muroňová, J., Lambert, E., Thamwan, C., Wehbe, Z., Court, M., Chevalier, G., Escoffier, J., Kherraf, Z. E., Coutton, C., Nef, S., Ray, P. F., Loeuillet, C., Martinez, G., & Arnoult, C. (2025). A comprehensive study of the sperm head defects in MMAF condition and their impact on embryo development in mice. Molecular |
| Nakamura S, Kobori Y, Ueda Y, Tanaka Y, Ishikawa H, Yoshida A, Katsumi M, Saito K, Nakamura A, Ogata T et al. STX2 is a causative gene for nonobstructive azoospermia. Hum Mutat 2018;39: 830-833. |
| Nie, X., & Arend, L. J. (2013). Pkd1 is required for male reproductive tract development. Mechanisms of Development, 130(11–12), 567–576. https://doi.org/10.1016/j.mod.2013.07.006 |
| Nozawa, K., Satouh, Y., Fujimoto, T., Oji, A., & Ikawa, M. (2018). Sperm-borne phospholipase C zeta-1 ensures monospermic fertilization in mice. Scientific reports, 8(1), 1315.https://doi.org/10.1038/s41598-018-19497-6 |
| O'Callaghan, E., Navarrete-Lopez, P., Štiavnická, M., Sánchez, J. M., Maroto, M., Pericuesta, E., Fernández-González, R., O'Meara, C., Eivers, B., Kelleher, M. M., Evans, R. D., Mapel, X. M., Lloret-Villas, A., Pausch, H., Balastegui-Alarcón, M., Avilés, M., Sanchez-Rodriguez, A., Roldan, E. R. S., McDonald, M., Kenny, D. |
| O'Donnell L, Rhodes D, Smith SJ, Merriner DJ, Clark BJ, Borg C, et al. An essential role for katanin p80 and microtubule severing in male gamete production. PLoS genetics. 2012; 8.https://doi.org/10.1371/journal.pgen.1002698 |
| O'Donnell L, McLachlan RI, Merriner DJ, O'Bryan MK, Jamsai D. KATNB 1 in the human testis and its genetic variants in fertile and oligoasthenoteratozoospermic infertile men. Andrology. 2014; 2: 884-91. https://doi.org/10.1111/andr.276 |
| Okutman, O., Muller, J., Baert, Y., Serdarogullari, M., Gultomruk, M., Piton, A., ... & Viville, S. (2015). Exome sequencing reveals a nonsense mutation in TEX15 causing spermatogenic failure in a Turkish family. Human molecular genetics, 24(19), 5581-5588.https://doi.org/10.1093/hmg/ddv290 |
| Oud MS, Okutman O¨, Hendricks LAJ, de Vries PF, Houston BJ, Vissers LELM, O'Bryan MK, Ramos L, Chemes HE, Viville S et al. Exome sequencing reveals novel causes as well as new candidate genes for human globozoospermia. Hum Reprod 2020;35:240-252.https://doi.org/10.1093/humrep/dez246 |
| Ounis, L., Zoghmar, A., Coutton, C., Rouabah, L., Hachemi, M., Martinez, D., ... & Ray, P. F. (2015). Mutations of the aurora kinase C gene causing macrozoospermia are the most frequent genetic cause of male infertility in Algerian men. Asian Journal of Andrology, 17(1), 68-73.https://doi.org/10.4103/1008- |
| Petukhova, G. V., Romanienko, P. J., & Camerini-Otero, R. D. (2003). The Hop2 protein has a direct role in promoting interhomolog interactions during mouse meiosis. Developmental cell, 5(6), 927-936.https://doi.org/10.1016/S1534-5807(03)00369-1 |
| Philipps DL, Wigglesworth K, Hartford SA, Sun F, Pattabiraman S, Schimenti K, et al. The dual bromodomain and WD repeat-containing mouse protein BRWD1 is required for normal spermiogenesis and the oocyte-embryo transition. Developmental biology. 2008; 317: 72- |
| Pierre V, Martinez G, Coutton C, Delaroche J, Yassine S, Novella C, et al. Absence of Dpy19l2, a new inner nuclear membrane protein, causes globozoospermia in mice by preventing the anchoring of the acrosome to the nucleus. Development. 2012; 139: 2955-65.https://doi.org/10.1242/dev.077982 |
| Qi, H., Moran, M. M., Navarro, B., Chong, J. A., Krapivinsky, G., Krapivinsky, L., Kirichok, Y., Ramsey, I. S., Quill, T. A., & Clapham, D. E. (2007). All four CatSper ion channel proteins are required for male fertility and sperm cell hyperactivated motility. Proceedings of the National Academy of Sciences of the United States |
| Qian X, Wang L, Zheng B, Shi Z-M, Ge X, Jiang C-F, et al. Deficiency of Mkrn2 causes abnormal spermiogenesis and spermiation, and impairs male fertility. Scientific reports. 2016; 6: 39318. https://doi.org/10.1038/srep39318 |
| Qiu, Y., Shimada, K., Yamamoto, K., & Ikawa, M. (2025). Loss of CCDC188 causes male infertility with defects in the sperm head-neck connection in mice†. Biology of reproduction, 112(1), 169-178. https://doi.org/10.1093/biolre/ioae137 |
| Ravel, C., Chantot-Bastaraud, S., El Houate, B., Berthaut, I., Verstraete, L., De Larouziere, V., Lourenço, D., Dumaine, A., Antoine, J. M., Mandelbaum, J., Siffroi, J. P., & McElreavey, K. (2007). Mutations in the protamine 1 gene associated with male infertility. Molecular human reproduction, 13(7), 461-464. |
| Riera-Escamilla, A., Enguita-Marruedo, A., Moreno-Mendoza, D., Chianese, C., Sleddens-Linkels, E., Contini, E., ... & Krausz, C. (2019). Sequencing of a 'mouse azoospermia'gene panel in azoospermic men: identification of RNF212 and STAG3 mutations as novel genetic causes of meiotic arrest. Human |

Rilianawati, Speed, R., Taggart, M., & Cooke, H. J. (2003). Spermatogenesis in testes of Dazl null mice after transplantation of wild-type germ cells. Reproduction, 126(5), 599–604.

Roy, A., Lin, Y. N., Agno, J. E., DeMayo, F. J., & Matzuk, M. M. (2009). Tektin 3 is required for progressive sperm motility in mice. Molecular Reproduction and Development, 76(5), 453–459. https://doi.org/10.1002/mrd.20957

Roy, A., Lin, Y.-N., Agno, J. E., DeMayo, F. J., & Matzuk, M. M. (2007). Absence of tektin 4 causes asthenozoospermia and subfertility in male mice. The FASEB Journal, 21, 1013–1025. https://doi.org/10.1096/fj.06-7035com

Sairam, M. R., & Krishnamurthy, H. (2001). The role of follicle-stimulating hormone in spermatogenesis: lessons from knockout animal models. Archives of medical research, 32(6), 601-608. https://doi.org/10.1016/s0188-4409(01)00328-9

Sampson, M. J., Decker, W. K., Beaudet, A. L., Ruitenbeek, W., Armstrong, D., Hicks, M. J., & Craigen, W. J. (2001). Immotile sperm and infertility in mice lacking mitochondrial voltage-dependent anion channel type 3. Journal of Biological Chemistry, 276(42), 39206–39212. https://doi.org/10.1074/jbc.M104724200

Santi, C. M., Martínez-López, P., de la Vega-Beltrán, J. L., Butler, A., Alisio, A., Darszon, A., & Salkoff, L. (2010). The SLO3 sperm-specific potassium channel plays a vital role in male fertility. FEBS Letters, 584(5), 1041–1046. https://doi.org/10.1016/j.febslet.2010.02.005

Sapiro, R., Kostetskii, I., Olds-Clarke, P., Gerton, G. L., Radice, G. L., & Strauss, J. F. III. (2002). Male infertility, impaired sperm motility, and hydrocephalus in mice deficient in sperm-associated antigen 6. Molecular and Cellular Biology, 22(17), 6298–6305. https://doi.org/10.1128/MCB.22.17.6298-6305.2002

Schilit, S. L. P., Menon, S., Friedrich, C., Kammin, T., Wilch, E., Hanscom, C., Jiang, S., Kliesch, S., Talkowski, M. E., Tüttelmann, F., MacQueen, A. J., & Morton, C. C. (2020). SYCP2 Translocation-Mediated Dysregulation and Frameshift Variants Cause Human Male Infertility. American journal of human genetics,

Seabra, C. M., Quental, S., Lima, A. C., Carvalho, F., Gonçalves, J., Fernandes, S., ... & Lopes, A. M. (2015). The mutational spectrum of WT1 in male infertility. The Journal of urology, 193(5), 1709-1715.https://doi.org/10.1016/j.juro.2014.11.004

Sha, Y., Liu, W., Li, S., Osadchuk, L. V., Chen, Y., Nie, H., Gao, S., Xie, L., Qin, W., Zhou, H., & Li, L. (2023). Deficiency in AK9 causes asthenozoospermia and male infertility by destabilising sperm nucleotide homeostasis. EBioMedicine, 96, 104798. https://doi.org/10.1016/j.ebiom.2023.104798

Sha, Y. W., Xu, X., Mei, L. B., Li, P., Su, Z. Y., He, X. Q., & Li, L. (2017). A homozygous CEP135 mutation is associated with multiple morphological abnormalities of the sperm flagella (MMAF). Gene, 633, 48–53.

Sha, Y., Liu, W., Zhu, X., Weng, M., Zhang, X., Wang, Y., & Zhou, H. (2021). Biallelic mutations of CFAP58 are associated with multiple morphological abnormalities of the sperm flagella. Clinical Genetics, 99(3), 443–448. https://doi.org/10.1111/cge.13898

Sha, Y., Liu, W., Tang, S., Zhang, X., Xiao, Z., Xiao, Y., Deng, H., Zhou, H., & Wei, X. (2023). TENT5D disruption causes oligoasthenoteratozoospermia and male infertility. Andrology, 11(6), 1121-1131. https://doi.org/10.1111/andr.13407

Sha, Y., Chen, Y., Wang, X., Meng, R., Yang, X., Li, Y., Jin, P., Li, S., Chen, J., Shao, T., Xu, D., Guo, Y., Jiang, Z., Li, Y., Yu, S., Li, L., & Wang, F. (2022). Biallelic mutations in IQCN, encoding a novel acroplaxome protein, lead to fertilization failure and male infertility with defects in the acrosome and shaping

Shang Y, Zhu F, Wang L, Ouyang Y-C, Dong M-Z, Liu C, et al. Essential role for SUN5 in anchoring sperm head to the tail. elife. 2017; 6: e28199. https://doi.org/10.7554/eLife.28199

Sha YW, Wang X, Su ZY, Wang C, Ji ZY, Mei LB, Zhang L, Deng BB, Huang XJ, Yan W et al. TDRD6 is associated with oligoasthenoteratozoospermia by sequencing the patient from a consanguineous family. Gene 2018;659:84-88https://doi.org/10.1016/j.gene.2018.03.040

Sha YW, Sha YK, Ji ZY, Mei LB, Ding L, Zhang Q, Qiu PP, Lin SB, Wang X, Li P et al. TSGA10 is a novel candidate gene associated with acephalic spermatozoa. Clin Genet 2018;93:776-783. https://doi.org/10.1111/cge.13140

Shen, Q., Martinez, G., Liu, H., Beurois, J., Wu, H., Amiri-Yekta, A., Liang, D., Kherraf, Z. E., Bidart, M., Cazin, C., Celse, T., Satre, V., Thierry-Mieg, N., Whitfield, M., Touré, A., Song, B., Lv, M., Li, K., Liu, C., Tao, F., He, X., Zhang, F., Arnoult, C., Ray, P. F., Cao, Y., & Coutton, C. (2021). Bi-allelic truncating

Shen, Y., Zhang, F., Li, F., Jiang, X., Yang, Y., Li, X., ... (2019). Loss-of-function mutations in QRICH2 cause male infertility with multiple morphological abnormalities of the sperm flagella. Nature Communications, 10, 1–15. https://doi.org/10.1038/s41467-018-08182-x

Shi, L., Zhou, T., Huang, Q., Zhang, S., Li, W., Zhang, L., Hess, R. A., Pazour, G. J., & Zhang, Z. (2019). Intraflagellar transport protein 74 is essential for spermatogenesis and male fertility in mice. Biology of Reproduction, 101(1), 188–199. https://doi.org/10.1093/biolre/ioz071

Shin, Y. H., Choi, Y., Erdin, S. U., Yatsenko, S. A., Kloc, M., Yang, F., ... & Rajkovic, A. (2010). Hormad1 mutation disrupts synaptonemal complex formation, recombination, and chromosome segregation in mammalian meiosis. PLoS genetics, 6(11), e1001190.https://doi.org/10.1371/journal.pgen.1001190

Shoji, M., Tanaka, T., Hosokawa, M., Reuter, M., Stark, A., Kato, Y., ... & Chuma, S. (2009). The TDRD9-MIWI2 complex is essential for piRNA-mediated retrotransposon silencing in the mouse male germline. Developmental cell, 17(6), 775-787.https://doi.org/10.1016/j.devcel.2009.10.012

Suryavathi V, Khattri A, Gopal K, Rani DS, Panneerdoss S, Gupta NJ, et al. Novel variants in UBE2B gene and idiopathic male infertility. Journal of andrology. 2008; 29: 564-71.https://doi.org/10.2164/jandrol.107.004580

Takasaki, N., Tachibana, K., Ogasawara, S., Matsuzaki, H., Hagiuda, J., Ishikawa, H., Mochida, K., Inoue, K., Ogonuki, N., Ogura, A., Noce, T., Ito, C., Toshimori, K., & Narimatsu, H. (2014). A heterozygous mutation of GALNTL5 affects male infertility with impairment of sperm motility. Proceedings of the

Tan, C., Meng, L., Lv, M., He, X., Sha, Y., Tang, D., Tan, Y., Hu, T., He, W., Tu, C., Nie, H., Zhang, H., Du, J., Lu, G., Fan, L. Q., Cao, Y., Lin, G., & Tan, Y. Q. (2022). Bi-allelic variants in DNHD1 cause flagellar axoneme defects and asthenoteratozoospermia in humans and mice. American Journal of Human Genetics,


Tan, C., Meng, L., Lv, M., He, X., Sha, Y., Tang, D., Tan, Y., Hu, T., He, W., Tu, C., Nie, H., Zhang, H., Du, J., Lu, G., Fan, L. Q., Cao, Y., Lin, G., & Tan, Y. Q. (2022). Bi-allelic variants in DNHD1 cause flagellar axoneme defects and asthenoteratozoospermia in humans and mice. American Journal of Human Genetics,

Tan, Y. Q., Tu, C., Meng, L., Yuan, S., Sjaarda, C., Luo, A., ... & Lin, G. (2019). Loss-of-function mutations in TDRD7 lead to a rare novel syndrome combining congenital cataract and nonobstructive azoospermia in humans. Genetics in Medicine, 21(5), 1209-1217.https://doi.org/10.1038/gim.2017.130

Tanaka, H., Iguchi, N., Toyama, Y., Kitamura, K., Takahashi, T., Kaseda, K., et al. (2004). Mice deficient in the axonemal protein Tektin-t exhibit male infertility and immotile-cilium syndrome due to impaired inner arm dynein function. Molecular and Cellular Biology, 24, 7958–7964.

Tanaka, T., Hosokawa, M., Vagin, V. V., Reuter, M., Hayashi, E., Mochizuki, A. L., ... & Chuma, S. (2011). Tudor domain containing 7 (Tdrd7) is essential for dynamic ribonucleoprotein (RNP) remodeling of chromatoid bodies during spermatogenesis. Proceedings of the National Academy of Sciences, 108(26),

Tang, S., Wang, X., Li, W., Yang, X., Li, Z., Liu, W., Li, C., Zhu, Z., Wang, L., Wang, J., Zhang, L., Sun, X., Zhi, E., Wang, H., Li, H., Jin, L., Luo, Y., Wang, J., Yang, S., & Zhang, F. (2017). Biallelic mutations in CFAP43 and CFAP44 cause male infertility with multiple morphological abnormalities of the sperm flagella.

Teng, Y. N., Chang, Y. P., Tseng, J. T., Kuo, P. H., Lee, I. W., Lee, M. S., & Kuo, P. L. (2012). A single-nucleotide polymorphism of the DAZL gene promoter confers susceptibility to spermatogenic failure in the Taiwanese Han. Human Reproduction, 27(9), 2857–2865. https://doi.org/10.1093/humrep/des227

Tian, H., Huo, Y., Zhang, J., Ding, S., Wang, Z., Li, H., ... & Zhang, Q. (2019). Disruption of ubiquitin specific protease 26 gene causes male subfertility associated with spermatogenesis defects in mice. Biology of Reproduction, 100(4), 1118-1128.https://doi.org/10.1093/biolre/ioy258

Touré, A., Lhuillier, P., Gossen, J. A., Kuil, C. W., Lhôte, D., Jégou, B., Escalier, D., & Gacon, G. (2007). The testis anion transporter 1 (Slc26a8) is required for sperm terminal differentiation and male fertility in the mouse. Human Molecular Genetics, 16(15), 1783–1793. https://doi.org/10.1093/hmg/ddm117

Tsai-Morris CH, Sheng Y, Lee E, Lei KJ, Dufau ML. Gonadotropinregulated testicular RNA helicase (GRTH/Ddx25) is essential for spermatid development and completion of spermatogenesis. Proc Natl Acad Sci USA 2004;101:6373-6378.https://doi.org/10.1073/pnas.0401855101

Tsai-Morris CH, Koh E, Sheng Y, Maeda Y, Gutti R, Namiki M, Dufau ML. Polymorphism of the GRTH/DDX25 gene in normal and infertile Japanese men: a missense mutation associated with loss of GRTH phosphorylation. Mol Hum Reprod 2007;13: 887-892https://doi.org/10.1093/molehr/gam065

Tsai-Morris CH, Sheng Y, Lee E, Lei KJ, Dufau ML. Gonadotropinregulated testicular RNA helicase (GRTH/Ddx25) is essential for spermatid development and completion of spermatogenesis. ProcNatl Acad Sci USA 2004;101:6373-6378.https://doi.org/10.1073/pnas.0401855101

Tu, C., Cong, J., Zhang, Q., He, X., Zheng, R., Yang, X., Gao, Y., Wu, H., Lv, M., Gu, Y., Lu, S., Liu, C., Tian, S., Meng, L., Wang, W., Tan, C., Nie, H., Li, D., Zhang, H., ... Tan, Y. Q. (2021). Bi-allelic mutations of DNAH10 cause primary male infertility with asthenoteratozoospermia in humans and mice. American

Tu, C., Nie, H., Meng, L., Yuan, S., He, W., Luo, A., Li, H., Li, W., Du, J., Lu, G., Lin, G., & Tan, Y. Q. (2019). Identification of DNAH6 mutations in infertile men with multiple morphological abnormalities of the sperm flagella. Scientific Reports, 9, 15864. https://doi.org/10.1038/s41598-019-52436-7

| |
|---|
| Valdes-Socin, H., Salvi, R., Daly, A. F., Gaillard, R. C., Quatresooz, P., Tebeu, P. M., Pralong, F. P., & Beckers, A. (2004). Hypogonadism in a patient with a mutation in the luteinizing hormone beta-subunit gene. The New England journal of medicine, 351(25), 2619-2625.https://doi.org/10.1056/NEJMoa040326 |
| Vasileva, A., Tiedau, D., Firooznia, A., Müller-Reichert, T., & Jessberger, R. (2009). Tdrd6 is required for spermiogenesis, chromatoid body architecture, and regulation of miRNA expression. Current Biology, 19(8), 630-639.https://doi.org/10.1016/j.cub.2009.02.047 |
| Wang, D., King, S. M., Quill, T. A., Doolittle, L. K., & Garbers, D. L. (2003). A new sperm-specific Na+/H+ exchanger required for sperm motility and fertility. Nature Cell Biology, 5(12), 1117–1122. https://doi.org/10.1038/ncb1072 |
| Wang, X., Shen, G., Yang, Y., Jiang, C., Ruan, T., Yang, X., Zhuo, L., Zhang, Y., Ou, Y., Zhao, X., Long, S., Tang, X., Lin, T., & Shen, Y. (2024). DNAH3 deficiency causes flagellar inner dynein arm loss and male infertility in humans and mice. eLife, 13, RP96755. https://doi.org/10.7554/eLife.96755 |
| Wang, W., Tian, S., Nie, H., Tu, C., Liu, C., Li, Y., Li, D., Yang, X., Meng, L., Hu, T., Zhang, Q., Du, J., Fan, L., Lu, G., Lin, G., Zhang, F., & Tan, Y. Q. (2021). CFAP65 is required in the acrosome biogenesis and mitochondrial sheath assembly during spermiogenesis. Human Molecular Genetics, 30(23), 2240–2254. |
| Wang, X. N., Li, Z. S., Ren, Y., Jiang, T., Wang, Y. Q., Chen, M., ... & Gao, F. (2013). The Wilms tumor gene, Wt1, is critical for mouse spermatogenesis via regulation of sertoli cell polarity and is associated with non-obstructive azoospermia in humans. PLoS genetics, 9(8). |
| Wang, X., Sha, Y. W., Wang, W. T., Cui, Y. Q., Chen, J., Yan, W., ... & Wang, J. (2019). Novel IFT140 variants cause spermatogenic dysfunction in humans. Molecular Genetics & Genomic Medicine, 7(9), e920. https://doi.org/10.1002/mgg3.920 |
| Wang, G., Zhu, X., Gao, Y., Lv, M., Li, K., Tang, D., Wu, H., Xu, C., Geng, H., Shen, Q., Zha, X., Duan, Z., Zhang, J., Hua, R., Tao, F., Zhou, P., Wei, Z., Cao, Y., Guo, R., & He, X. (2022). Biallelic loss-of-function mutations in SEPTIN4 (C17ORF47), encoding a conserved annulus protein, cause thin midpiece spermatozoa |
| Wang, J., Jin, H. J., Lu, Y., Wang, Z. H., Li, T. Y., Xia, L., Li, H. J., Wang, B. B., & Chen, S. R. (2024). Discovery of CCDC188 gene as a novel genetic target for human acephalic spermatozoa syndrome. Protein & cell, 15(9), 704-709. https://doi.org/10.1093/procel/pwae018 |
| Wedenoja, S., Khamaysi, A., Shimshilashvili, L., Anbtawe-Jomaa, S., Elomaa, O., Toppari, J., Höglund, P., Aittomäki, K., Holmberg, C., Hovatta, O., Tapanainen, J. S., Ohana, E., & Kere, J. (2017). A missense mutation in SLC26A3 is associated with human male subfertility and impaired activation of CFTR. Scientific |
| Williams, H. L., Mansell, S., Alasmari, W., Brown, S. G., Wilson, S. M., Sutton, K. A., Miller, M. R., Lishko, P. V., Barratt, C. L., Publicover, S. J., & Martins da Silva, S. (2015). Specific loss of CatSper function is sufficient to compromise fertilizing capacity of human spermatozoa. Human Reproduction, 30(12), |
| Won, J., Marín de Evsikova, C., Smith, R. S., Hicks, W. L., Edwards, M. M., Longo-Guess, C., Li, T., Naggert, J. K., & Nishina, P. M. (2011). NPHP4 is necessary for normal photoreceptor ribbon synapse maintenance and outer segment formation, and for sperm development. Human Molecular Genetics, 20(3), |
| Wu, W. B., Li, Y. S., Ji, X. F., Wang, Q. X., Gao, X. M., Yang, X. F., Pan, Z. H., & Feng, X. X. (2012). [Expression of TEKT4 protein decreases in the ejaculated spermatozoa of idiopathic asthenozoospermic men]. Zhonghua Nan Ke Xue, 18(6), 514–517. (In Chinese) |
| Wu, H., Liu, Y., Li, Y., Li, K., Xu, C., Gao, Y., Lv, M., Guo, R., Xu, Y., Zhou, P., Wei, Z., Hua, R., He, X., & Cao, Y. (2023). DNALI1 deficiency causes male infertility with severe asthenozoospermia in humans and mice by disrupting the assembly of the flagellar inner dynein arms and fibrous sheath. Cell Death & Disease, |
| Wu, Y., Li, Y., Murtaza, G., Zhou, J., Jiao, Y., Gong, C., Hu, C., Han, Q., Zhang, H., Zhang, Y., Shi, B., Ma, H., Jiang, X., & Shi, Q. (2021). Whole-exome sequencing of consanguineous families with infertile men and women identifies homologous mutations in SPATA22 and MEIOB. Human Reproduction, 36(10), 2793- |
| Xiao N, Kam C, Shen C, Jin W, Wang J, Lee KM, et al. PICK1 deficiency causes male infertility in mice by disrupting acrosome formation. The Journal of clinical investigation. 2009; 119: 802-12. https://doi.org/10.1172/JCI36230 |
| Xin, A., Qu, R., Chen, G., Zhang, L., Chen, J., Tao, C., Fu, J., Tang, J., Ru, Y., Chen, Y., Peng, X., Shi, H., Zhang, F., & Sun, X. (2020). Disruption in ACTL7A causes acrosomal ultrastructural defects in human and mouse sperm as a novel male factor inducing early embryonic arrest. Science Advances, 6(35), eaaz4796. |
| Xu, C., Tang, D., Shao, Z., Geng, H., Gao, Y., Li, K., Tan, Q., Wang, G., Wang, C., Wu, H., Li, G., Lv, M., He, X., & Cao, Y. (2022). Homozygous SPAG6 variants can induce nonsyndromic asthenoteratozoospermia with severe MMAF. Reproductive Biology and Endocrinology, 20(1), 41. https://doi.org/10.1186/s12958-022- |
| Xu, K., Yang, L., Zhang, L., & Qi, H. (2020). Lack of AKAP3 disrupts integrity of the subcellular structure and proteome of mouse sperm and causes male sterility. Development, 147(2), dev181057. https://doi.org/10.1242/dev.181057 |

Xu, X., Sha, Y. W., Mei, L. B., Ji, Z. Y., Qiu, P. P., Ji, H., Li, P., Wang, T., & Li, L. (2018). A familial study of twins with severe asthenozoospermia identified a homozygous SPAG17 mutation by whole-exome sequencing. Clinical Genetics, 93(2), 345–349. https://doi.org/10.1111/cge.13059

Yamazaki, D., Miyata, H., Funato, Y., Fujihara, Y., Ikawa, M., & Miki, H. (2016). The $Mg^{2+}$ transporter CNNM4 regulates sperm $Ca^{2+}$ homeostasis and is essential for reproduction. Journal of Cell Science, 129(9), 1940–1949. https://doi.org/10.1242/jcs.182220

Yan, W., Si, Y., Slaymaker, S., Li, J., Zheng, H., Young, D. L., ... & Charo, I. F. (2010). Zmynd15 encodes a histone deacetylase-dependent transcriptional repressor essential for spermiogenesis and male fertility. Journal of Biological Chemistry, 285(41), 31418-31426.https://doi.org/10.1074/jbc.M110.116418

Yang, F., Silber, S., Leu, N. A., Oates, R. D., Marszalek, J. D., Skaletsky, H., ... Wang, P. J. (2015). TEX11 is mutated in infertile men with azoospermia and regulates genome-wide recombination rates in mouse. EMBO Molecular Medicine, 7(9), 1198–1210. https://doi.org/10.15252/emmm.201404967

Yang, F., Eckardt, S., Leu, N. A., McLaughlin, K. J., & Wang, P. J. (2008). Mouse TEX15 is essential for DNA double-strand break repair and chromosomal synapsis during male meiosis. Journal of Cell Biology, 180(4), 673-679. https://doi.org/10.1083/jcb.200709057

Yang, K. T., Li, S. K., Chang, C. C., Tang, C. J. C., Lin, Y. N., Lee, S. C., & Tang, T. K. (2010). Aurora-C kinase deficiency causes cytokinesis failure in meiosis I and production of large polyploid oocytes in mice. Molecular biology of the cell, 21(14), 2371-2383.https://doi.org/10.1091/mbc.e10-02-0170

Yang, X., Liu, F., Li, J. et al. Association study of protamine 2 (PRM2) gene polymorphism with male infertility in Chinese Han population. Genes Genom 38, 311-317 (2016).https://doi.org/10.1007/s13258-015-0368-0

Yang, F., De La Fuente, R., Leu, N. A., Baumann, C., McLaughlin, K. J., & Wang, P. J. (2006). Mouse SYCP2 is required for synaptonemal complex assembly and chromosomal synapsis during male meiosis. The Journal of cell biology, 173(4), 497-507.https://doi.org/10.1083/jcb.200603063

Yatsenko, A. N., Georgiadis, A. P., Röpke, A., Berman, A. J., Jaffe, T., Olszewska, M., ... Tüttelmann, F. (2015). X-linked TEX11 mutations, meiotic arrest, and azoospermia in infertile men. New England Journal of Medicine, 372(22), 2097–2107. https://doi.org/10.1056/NEJMoa1406192

Yatsenko, A. N., O'Neil, D. S., Roy, A., Arias-Mendoza, P. A., Chen, R., Murthy, L. J., ... & Matzuk, M. M. (2012). Association of mutations in the zona pellucida binding protein 1 (ZPBP1) gene with abnormal sperm head morphology in infertile men. Molecular human reproduction, 18(1), 14-21.

Ye, J. W., Abbas, T., Zhou, J. T., Chen, J., Yang, M. L., Huang, X. H., Zhang, H., Ma, H., Ma, A., Xu, B., Murtaza, G., Shi, Q. H., & Shi, B. L. (2024). Homozygous CCDC146 mutation causes oligoasthenoteratozoospermia in humans and mice. Zoological Research, 45(5), 1073–1087.

Yu, Y., Wang, J., Zhou, L., Li, H., Zheng, B., & Yang, S. (2021). CFAP43-mediated intra-manchette transport is required for sperm head shaping and flagella formation. Zygote, 29(1), 75–81. https://doi.org/10.1017/S0967199420000556

Zhang, X., Xiao, Z., Zhang, J., Xu, C., Liu, S., Cheng, L., Zhou, S., Zhao, S., Zhang, Y., Wu, J., Wang, Y., & Liu, M. (2022). Differential requirements of IQUB for the assembly of radial spoke 1 and the motility of mouse cilia and flagella. Cell Reports, 41(8), 111683. https://doi.org/10.1016/j.celrep.2022.111683

Zhang, Z., Zhou, H., Deng, X., Zhang, R., Qu, R., Mu, J., Liu, R., Zeng, Y., Chen, B., Wang, L., Sang, Q., & Bao, S. (2023). IQUB deficiency causes male infertility by affecting the activity of p-ERK1/2/RSPH3. Human Reproduction, 38(1), 168–179. https://doi.org/10.1093/humrep/deac244

Zhang, G., Li, D., Tu, C., Meng, L., Tan, Y., Ji, Z., Cheng, J., Lu, G., Lin, G., Zhang, H., et al. (2021). Loss-of-function missense variant of AKAP4 induced male infertility through reduced interaction with QRICH2 during sperm flagella development. Human Molecular Genetics, 31, 219–231.

Zhang, X., Zheng, R., Liang, C., Liu, H., Zhang, X., Ma, Y., Liu, M., Zhang, W., Yang, Y., Liu, M., Jiang, C., Ren, Q., Wang, Y., Chen, S., Yang, Y., & Shen, Y. (2022). Loss-of-function mutations in CEP78 cause male infertility in humans and mice. Science Advances, 8(40), eabn0968. https://doi.org/10.1126/sciadv.abn0968

Zhang, J., He, X., Wu, H., Zhang, X., Yang, S., Liu, C., Liu, S., Hua, R., Zhou, S., Zhao, S., Hu, F., Zhang, J., Liu, W., Cheng, H., Gao, Y., Zhang, F., Cao, Y., & Liu, M. (2021). Loss of DRC1 function leads to multiple morphological abnormalities of the sperm flagella and male infertility in human and mouse. Human

Zhang, B., Khan, I., Liu, C., Ma, A., Khan, A., Zhang, Y., ... (2021). Novel loss-of-function variants in DNAH17 cause multiple morphological abnormalities of the sperm flagella in humans and mice. Clinical Genetics, 99, 176–186. https://doi.org/10.1111/cge.13866

Zhang, W., Song, X., Ni, F., Cheng, J., Wu, B. L., & Jiang, H. (2017). Association analysis between HFM1 variations and idiopathic azoospermia or severe oligozoospermia in Chinese Men. Science China. Life Sciences, 60(3), 315.https://doi.org/10.1007/s11427-016-0274-9

Zhang, Y., Liu, H., Li, W., Zhang, Z., Zhang, S., Teves, M. E., ... & Zhang, Z. (2018). Intraflagellar transporter protein 140 (IFT140), a component of IFT-A complex, is essential for male fertility and spermiogenesis in mice. Cytoskeleton, 75(2), 70-84.https://doi.org/10.1002/cm.21427

Zhang, X., Huang, G., Jiang, T., Meng, L., Li, T., Zhang, G., Wu, N., Chen, X., Zhao, B., Li, N., Wu, S., Guo, J., Zheng, R., Ji, Z., Xu, Z., Wang, Z., Deng, D., Tan, Y., & Xu, W. (2024). CEP112 coordinates translational regulation of essential fertility genes during spermiogenesis through phase separation in humans and mice.

Zheng, H., Gong, C., Li, J., Hou, J., Gong, X., Zhu, X., Deng, H., Wu, H., Zhang, F., Shi, Q., Zhou, J., Shi, B., Yang, X., & Xi, Y. (2024). CCDC157 is essential for sperm differentiation and shows oligoasthenoteratozoospermia-related mutations in men. J Cell Mol Med, 28(7).

Zhou, D., Wu, H., Wang, L., Wang, X., Tang, S., Zhou, Y., Wang, J., Wu, B., Tang, J., Zhou, X., Tian, S., Liu, S., Lv, M., He, X., Jin, L., Shi, H., Zhang, F., Cao, Y., & Liu, C. (2024). Deficiency of MFSD6L, an acrosome membrane protein, causes oligoasthenoteratozoospermia in humans and mice. Journal of Genetics

Zhu, T., Zhang, Y., Sheng, X., Zhang, X., Chen, Y., Zhu, H., Guo, Y., Qi, Y., Zhao, Y., Zhou, Q., Chen, X., Guo, X., & Zhao, C. (2023). Absence of CEP78 causes photoreceptor and sperm flagella impairments in mice and a human individual. eLife, 12, e76157. https://doi.org/10.7554/eLife.76157

Zhu, Z. J., Wang, Y. Z., Wang, X. B., Yao, C. C., Zhao, L. Y., Zhang, Z. B., Wu, Y., Chen, W., & Li, Z. (2022). Novel mutation in ODF2 causes multiple morphological abnormalities of the sperm flagella in an infertile male. Asian Journal of Andrology, 24(5), 463–472. https://doi.org/10.4103/aja202183

Zhuang, B. J., Xu, S. Y., Dong, L., Zhang, P. H., Zhuang, B. L., Huang, X. P., Li, G. S., You, Y. D., Chen, D., Yu, X. J., & Chang, D. G. (2022). Novel DNAH1 mutation loci lead to multiple morphological abnormalities of the sperm flagella and literature review. World Journal of Men's Health, 40(4), 551–560.

Zhu F, Wang F, Yang X, Zhang J, Wu H, Zhang Z, et al. Biallelic SUN5 mutations cause autosomal-recessive acephalic spermatozoa syndrome. The American Journal of Human Genetics. 2016; 99: 942-9. https://doi.org/10.1016/j.ajhg.2016.08.004

Zhu F, Liu C, Wang F, Yang X, Zhang J, Wu H, et al. Mutations in PMFBP1 cause acephalic spermatozoa syndrome. The American Journal of Human Genetics. 2018; 103: 188-99.https://doi.org/10.1016/j.ajhg.2018.06.010

Zuccarello, D., Ferlin, A., Garolla, A., Pati, M. A., Moretti, A., Cazzadore, C., Francavilla, S., & Foresta, C. (2008). A possible association of a human tektin-t gene mutation (A229V) with isolated non-syndromic asthenozoospermia: Case report. Human Reproduction, 23(4), 996–1001.
